# Under-ice availability of phytoplankton lipids is key to freshwater zooplankton winter survival

**DOI:** 10.1038/s41598-017-10956-0

**Published:** 2017-09-14

**Authors:** Guillaume Grosbois, Heather Mariash, Tobias Schneider, Milla Rautio

**Affiliations:** 1 Department of Fundamental Sciences and Group for Interuniversity Research in Limnology and aquatic environment (GRIL), Université du Québec à Chicoutimi, Saguenay, Québec, Canada; 2 National Wildlife Research Centre, Environment and Climate Change Canada, Ottawa, Ontario, Canada

## Abstract

Shortening winter ice-cover duration in lakes highlights an urgent need for research focused on under-ice ecosystem dynamics and their contributions to whole-ecosystem processes. Low temperature, reduced light and consequent changes in autotrophic and heterotrophic resources alter the diet for long-lived consumers, with consequences on their metabolism in winter. We show in a survival experiment that the copepod *Leptodiaptomus minutus* in a boreal lake does not survive five months under the ice without food. We then report seasonal changes in phytoplankton, terrestrial and bacterial fatty acid (FA) biomarkers in seston and in four zooplankton species for an entire year. Phytoplankton FA were highly available in seston (2.6 µg L^−1^) throughout the first month under the ice. Copepods accumulated them in high quantities (44.8 µg mg dry weight^−1^), building lipid reserves that comprised up to 76% of body mass. Terrestrial and bacterial FA were accumulated only in low quantities (<2.5 µg mg dry weight^−1^). The results highlight the importance of algal FA reserve accumulation for winter survival as a key ecological process in the annual life cycle of the freshwater plankton community with likely consequences to the overall annual production of aquatic FA for higher trophic levels and ultimately for human consumption.

## Introduction

Winter is the most unexplored season in ecology and has often been portrayed as a dormant period for aquatic organisms especially if the ecosystem is ice-covered^1^. However, it is increasingly understood that critical ecological processes do not only take place under the ice but also determine the following summer season^2^. Several studies have reported winter-active zooplankton in lakes with evidence from both copepods and cladocerans growing and reproducing under the ice^3, 4^. However, the phytoplankton resource pool for zooplankton in the winter months preceding the spring bloom is limited^5^ and as a consequence, the challenge for winter-active zooplankton is to cope with the general lack of autotrophic food sources. Therefore, it is believed that zooplankton require alternative energy sources during the ice-covered months.

Because snow and ice covers drastically reduce incoming light^6^ for photosynthesis, phytoplankton, benthic algae and macrophytes are thought to be replaced by heterotrophic resources in winter. An increasing number of studies have suggested that allochthonous carbon inputs and microbial loop based bacterial production subsidize zooplankton in winter^7^. Indeed, the share of terrestrial carbon in zooplankton biomass, referred to as allochthony, reaches considerable quantities (>50%) in a large number of species and ecosystems and is related to the composition of organic matter sources among lakes^8^. It thus appears plausible to assume that on a seasonal scale, zooplankton reliance on heterotrophic sources would be highest during periods when aquatic primary production is low, as is the case in winter^9^.

However, zooplankton also have the ability to efficiently store energy by accumulating lipid reserves in late fall and early winter^4^. This is a well-documented strategy in polar marine copepods^10^, but knowledge in freshwater systems is scarce (but see Hiltunen *et al*.^11^ and Mariash *et al*.^12^). Lipids are highly energetic molecules as compared to both carbohydrates and proteins^13^. Lipid reserves in consumers are believed to be mostly derived from autochthonous food sources such as phytoplankton in pelagic environment, which are characterized by their high content of polyunsaturated fatty acids (PUFA). PUFA are a major component of lipids that are considered high quality food^14^. However, in a majority of allochthony studies based on stable isotopes, these lipid reserves have not been taken into account^15, 16^ and sometimes have been chemically removed^17^, as they are not considered indicators of the recent diet but have potentially been accumulated over a long time period^18^. They are thus not considered as part of baseline metabolism, but rather as latent energy reserves to be available for future metabolic requirements, enabling consumers to survive and reproduce during periods of food scarcity^4^. In many marine copepods, lipids are exhausted over the course of winter, suggesting that they have an important metabolic role under the ice^13, 19^. Interestingly, the highest copepod lipid content also in lakes has been measured during winter^18^ precisely when phytoplankton resources are the scarcest in the environment. During this time, dissolved organic carbon (DOC) of terrestrial origin is abundant in boreal lakes^20^. Although it cannot be directly consumed by zooplankton^21^, DOC may be assimilated in the microbial loop and trophically upgraded by heterotrophic protists^22^, thus constituting a potential resource for zooplankton to build lipid reserves or fuel day-to-day metabolism in an environment with low primary production and phytoplankton abundance.

On-going climate warming leads to the mobilization of terrestrial carbon pools, thereby increasing allochthonous carbon inputs from catchments into surface waters worldwide^23^. The fate of this terrestrial pool, including its use in sustaining secondary production of plankton and higher trophic levels in lakes is still largely debated^15, 24^. Many studies demonstrated a significant contribution of terrestrial organic matter to the biomass^8, 16^. However, whether allochthonous carbon sources contribute to the lipid reserve accumulation of zooplankton in winter, and to subsequent metabolic investment to growth, is not known. This information would contribute to our understanding of the roles, and relative importance of, autochthonous and allochthonous carbon sources in sustaining aquatic food webs.

The composition of lipid reserves differs depending on whether their origin is phytoplanktonic, bacterial or terrestrial. While same FA can be synthesized by numerous different species, aquatic food webs are characterized by a high abundance of long-chain n-3 PUFA^25^ with a strong prevalence of eicosapentaenoic acid (EPA) and docosahexaenoic acid (DHA) while terrestrial ecosystems have a dominance of n-6 PUFA and higher linoleic acid (LNA) and alpha-linolenic acid (ALA) contents^26^. Moreover, several FA are typically synthesized by terrestrial plants, whereas others are only found in aquatic primary producers^27^ and in many cases may be attributed to specific taxa^28, 29^. Long-chain saturated fatty acids (LC-SAFA, i.e. C20:0, C22:0, C24:0) are associated with terrestrial plants^30^ and are abundant in terrestrial leaves e.g. senescent beech leaves contain >40% of SAFA^31^. LC-SAFA are characteristic to various terrestrial plant species from temperate and boreal biomes, including birch, alder, cottonwood, maple and willow but they are common also in reed and peat^26, 32^. Other FA, such as branched and odd numbered FA (iso- and anteiso-C15:0 and C17:0) are biomarkers of bacteria^33^. Among PUFA, eicosapentaenoic acid (EPA, 20:5n-3), docosahexaenoic acid (DHA, 22:6n-3) and arachidonic acid (ARA, 20:4n-6) are mainly synthesized by algae^34^ and are considered essential molecules needed by zooplankton for growth, reproduction and regulation of membrane fluidity. Aquatic organisms have limited capacity to synthesize them *de novo* and thus need to acquire these specific PUFA mainly from their diet^35^. Decreasing algal production is therefore believed to influence the PUFA availability in the environment. This poses a challenge for winter-reproducing species to produce eggs^36^ and winter-active species to maintain membrane fluidity^37^, which requires PUFA, although PUFA are at lowest availability.

To better understand how phytoplanktonic, terrestrial and bacterial compounds contribute to zooplankton lipid reserves and how they are used by zooplankton in the course of the year and particularly during winter, we designed a study combining a laboratory survival experiment with a 12-month field survey. First, we experimentally estimated how long accumulated lipid reserves can sustain copepod metabolism. These results were then completed by a one-year lake sampling, where we measured in detail the phytoplanktonic, terrestrial and bacterial fatty acid availability in seston and their accumulation in four species of copepods and cladocerans. Since many zooplankton are active in winter under the ice and use lipid reserves mainly for reproduction^4^, we hypothesized that terrestrial organic matter and/or bacteria would be used as energy sources to maintain zooplankton in the absence of primary production. To test this hypothesis, we followed the survival of a calanoid copepod *Leptodiaptomus minutus* during winter without food and with winter seston diet, and expected survival to be higher when the copepods had access to winter seston presumed to be dominated by heterotrophic resources. We also hypothesised that terrestrial and bacterial fatty acids contribute to the lipid reserves according to their abundance in the seston, and that their contribution in the copepod lipid reserves would be highest in winter.

## Results

### Seasonal abundances in zooplankton community

**Figure 1:**
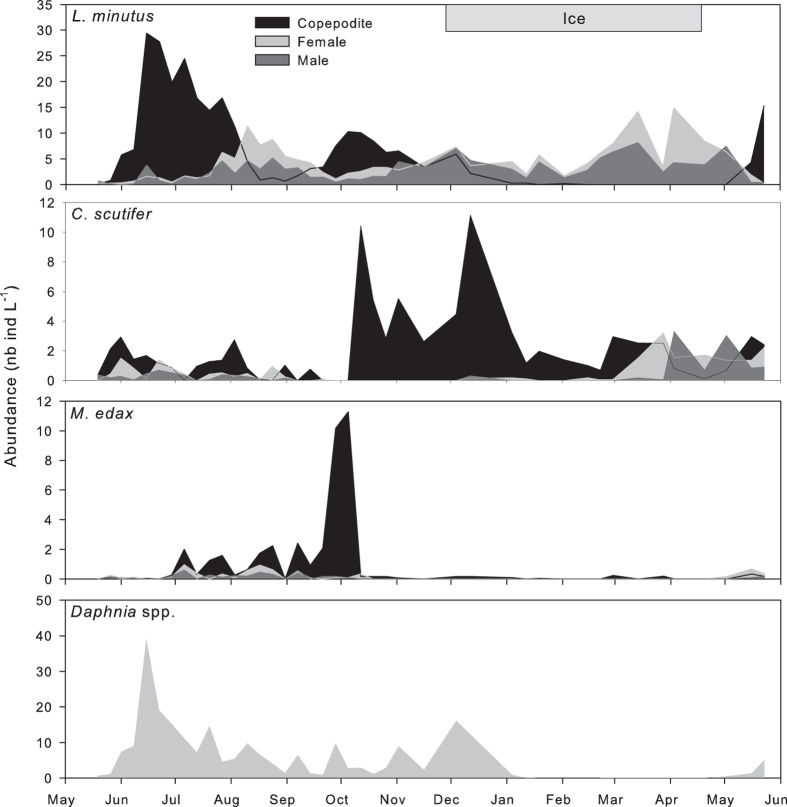
Seasonal abundance (individuals L^−1^) of copepodites (copepods), adult females and males of the four main species in the zooplankton community of Lake Simoncouche. *Daphnia* spp. numbers include females and males. Note the different scales.

*L. minutus*, *Cyclops scutifer* and *Daphnia* spp. were active during the whole year (Fig. 1). They were also the most abundant zooplankton in the lake with average annual abundances of 12.9 ± 4.9, 3.3 ± 1.7 and 4.9 ± 2.8 ind L^−1^ (mean ± SD), respectively. They represented 58%, 15% and 22% of the total community abundance. *L. minutus* spent the winter as an adult, but the *C. scutifer* population was entirely made of copepodite (C-IV) individuals from November to April. The *Daphnia* population was comprised of adults under the ice, with relatively high abundances until 12 December and then rather low until mid-May. No other copepods or cladocerans were present in the water column during winter except highly abundant *Bosmina* spp. in early winter and punctual presences of *Eucyclops speratus* and *Tropocyclops prasinus*. The cyclopoid *Mesocyclops edax* was absent from the water column from November to May although being the fourth most abundant crustacean zooplankton in the lake (1.0 ± 1.1 ind L^−1^ and 5% of the average annual community abundance).

### Starvation experiment

**Figure 2:**
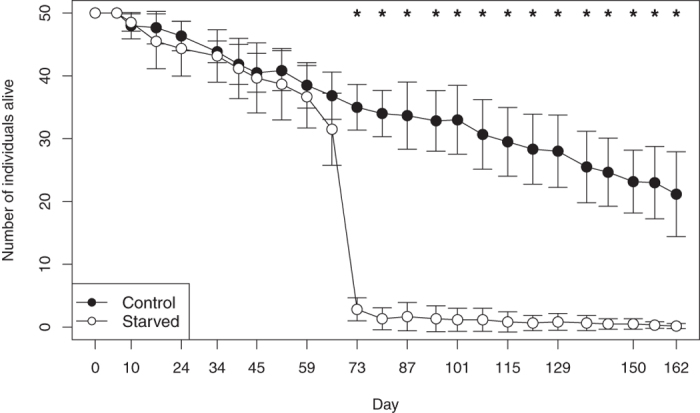
Adult *L. minutus* survival in the starvation experiment for individuals that were collected from the Lake Simoncouche when the lake was freezing in November (20-Nov-2012). Values are means of six replicates and SD. The experiment was terminated when the lake became ice-free in May (3-May-2013). The asterisks mark the days when the survival differed significantly among treatments.

The survival of *L. minutus* at first followed the same pattern in both treatments but after ten weeks started to differ strongly between starved and fed conditions (Fig. 2). Access to food significantly increased zooplankton survival (F_(1,245)_ = 1682.6, p < 0.0001) which was affected by time (F_(24,245)_ = 230.3, p < 0.0001) and both factors interacted (F_(24,245)_ = 48.7, p < 0.0001). During the first 66 days of the experiment, the survival was not significantly different among the treatments (p > 0.13; see Supplementary Table S2). Only from day 66 (29 January) to day 73 (5 February) the number of individuals alive in the starved condition dropped from 32 ± 6 to 3 ± 2 (mean ± SD; W = 36, p = 0.005). At the end of the experiment on day 163 (5 May), only 0.2 ± 0.4 starved individuals were still alive (i.e., one individual in one replicate), in contrast to 21 ± 7 surviving individuals with access to food.

### Seasonality in water chemistry and putative food sources

The most pronounced change in water chemistry, followed by shifts in putative food sources, took place in autumn. The most important increase in nutrient concentrations was registered from September to October (Table 1) when TDN almost doubled (×1.8) and TDP more than doubled (×2.2). DOC increased a month before, reaching its maximum already in September (6.6 mg C L^−1^). SUVA_254_ was lowest in August (3.4 L mg^−1^ m^−1^), increased in September (4.1 L mg^−1^ m^−1^) followed by lower values in winter (3.7 ± 0.1 L mg^−1^ m^−1^), and then reached its maximum in the end of March (4.5 L mg^−1^ m^−1^). Chl-a concentration was highest in July (3.5 µg L^−1^), decreased to a minimum in December (0.2 µg L^−1^) and remained very low throughout the winter, from December until March (0.4 ± 0.2 µg L^−1^). Bacterial biomass was highest in October (72.4 µg C L^−1^) and decreased in winter to the minimum value of 30.2 µg C L^−1^ in January (Table 1).

**Table 1:** Environmental and biological variables from the epilimnion of Lake Simoncouche represented by temperature (Temp, °C), total dissolved nitrogen (TDN, mg N L^−1^), total dissolved phosphorus (TDP, µg P L^−1^),dissolved organic carbon (DOC, mg C L^−1^), chlorophyll-a (Chl-a, µg L^−1^), bacteria biomass (Bact Biom, µg C L^−1^) and SUVA_254nm_ (L mg^−1^ m^−1^).

Date	Temp	TDN	TDP	DOC	Chl-a	Bact Biom	SUVA_254nm_
12-May-11	5.8	0.20	12.6	4.6	1.9	27.4	4.0
15-Jun-11	17.5	NA	NA	4.6	3.0	39.0	4.1
05-Jul-11	22.8	0.21	5.8	4.9	3.5	62.6	3.6
10-Aug-11	22.0	0.13	3.5	5.3	3.3	48.6	3.4
08-Sep-11	17.3	0.12	4.0	6.6	2.6	58.0	4.1
19-Oct-11	10.8	0.22	8.6	5.8	1.4	72.4	3.9
04-Dec-11	2.9	0.22	5.1	5.6	0.2	52.1	3.6
12-Jan-12	3.7	0.24	4.6	5.4	0.7	30.2	3.8
22-Feb-12	3.9	0.18	5.8	5.3	0.3	35.5	3.7
28-Mar-12	3.2	0.21	5.6	5.1	0.3	57.6	4.5
16-May-12	14.3	0.12	2.7	4.6	1.8	43.3	4.3

**Figure 3:**
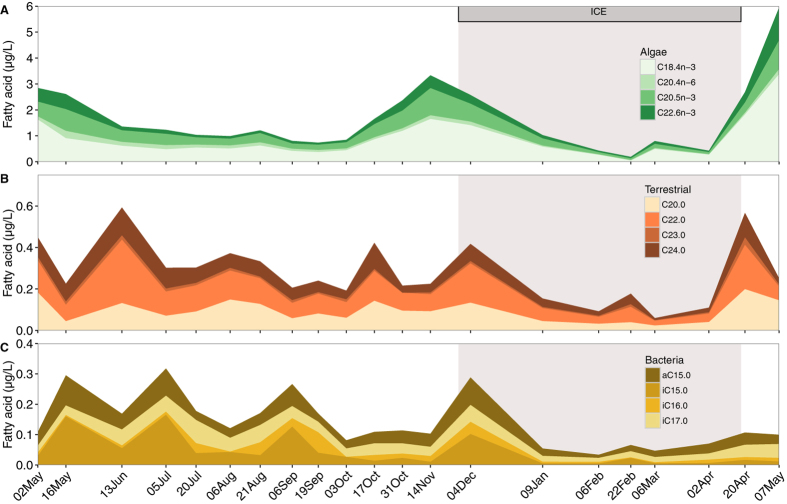
Seasonal pattern of seston fatty acids divided by FA biomarkers that represent phytoplankton, terrestrial and bacterial sources. Note the different scales on the Y-axis. Grey shade represents the period when the lake was ice covered. The sampling dates are indicated on the X-axis. FA with negligible contribution are not displayed.

The six phytoplankton FA biomarkers (FAB) dominated the seston FAB composition with an annual average of 1.7 ± 1.3 µg L^−1^ (mean ± SD), followed by terrestrial (0.3 ± 0.1 µg L^−1^) and bacterial FAB (0.2 ± 0.1 µg L^−1^) (Fig. 3). When expressed as a percentage of total FA in seston, the distribution was the following: 8.6% (±5.6) phytoplankton FAB, 1.5% (±0.9) terrestrial FAB and 1.2% (±1.3) bacterial FAB. All FAB of the three potential energy sources were more abundant in summer than in winter. Phytoplankton FAB had an average concentration of 0.8 µg L^−1^ under the ice and 2.1 µg L^−1^ in the ice-free period (t = −2.25, p = 0.04). The same pattern was observed for terrestrial biomarkers with 0.2 µg L^−1^ in winter and 0.3 µg L^−1^ in summer (t = −2.77, p = 0.02) as well as for bacterial biomarkers with 0.1 µg L^−1^ in winter and 0.2 µg L^−1^ in summer, although this difference was not significant (t = −1.99, p = 0.06).

### Total lipids and fatty acid composition in zooplankton

A broad range of lipid content was observed in copepods, especially in *L. minutus*, whose lipid content ranged from 18% (of dry weight) in mid-September to 76% in late January (see Supplementary Fig. S1). *C. scutifer* had its lowest lipid content (21%) in May and highest (50%) in January. The late January maximum of lipids for *L. minutus* and *C. scutifer* revealed that these copepods had accumulated lipids under the ice, while the decrease in their lipid content was measured starting only at the end of February. The lipid content of *M. edax* was stable around 28 ± 9% (mean ± SD) with a maximum of 47% in May. The lipid content of *Daphnia* was highest in winter (33% in February) although it remained relatively stable throughout the year (22 ± 5%).

**Figure 4:**
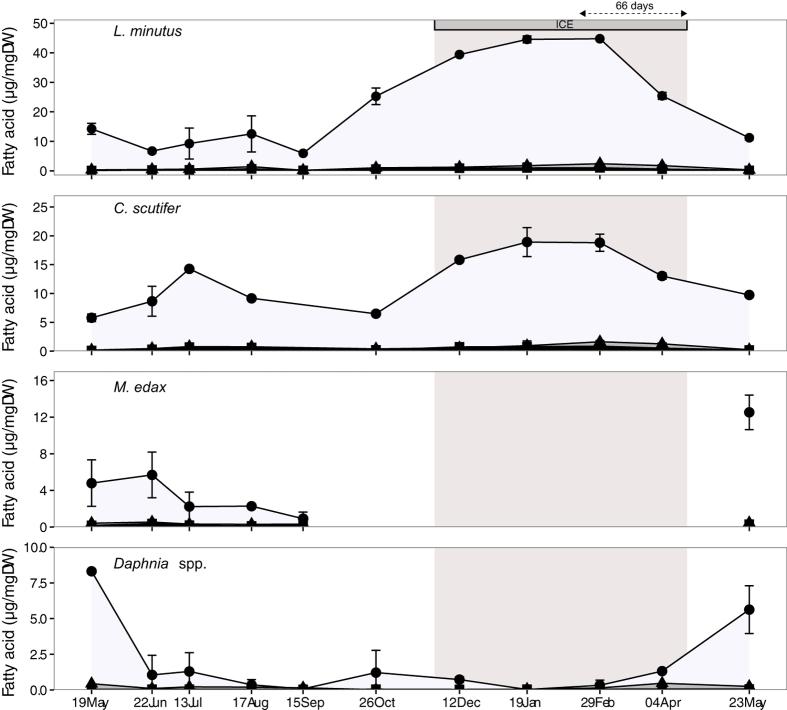
Cumulated concentration of fatty acid biomarkers of phytoplankton (filled circle), bacteria (filled triangle) and terrestrial (filled square) in zooplankton. The gap in *M. edax* represents a period of the year when all individuals were absent of the pelagic environment. Note the different scales on the Y-axis. Grey shade represents the period when the lake was ice covered.

The relative ranking of FAB was similar in *L. minutus*, *C. scutifer*, *M. edax* and *Daphnia* spp. (Fig. 4). Terrestrial FAB were present in concentrations between 0.05 and 0.5 µg mg dry weight^−1^ (µg mg DW^−1^) representing 0.5 to 1.3% of total FA, and bacterial FAB in similarly low concentrations between 0.2–1.0 µg mg DW^−1^ (1.2–1.9% of total FA). The most accumulated FAB were from phytoplankton, with an annual mean of 20.9 (40%), 12.4 (33%), 4.7 (19%) and 1.6 µg mg DW^−1^ (10%) for *L. minutus*, *C. scutifer*, *M. edax* and *Daphnia* spp., respectively. *L. minutus* contained more phytoplankton FAB under the ice (38.5 µg mg DW^−1^) than during the ice-free season (12.2 µg mg DW^−1^) (t = 5.1, p = 0.004). The same was true for terrestrial and bacterial FAB (p < 0.01). Likewise, *C. scutifer* contained more phytoplankton FAB under the ice (15.9 µg mg DW^−1^) than during the ice-free season (9.0 µg mg DW^−1^) (t = 3.32, p = 0.03), but with no significant difference among seasons for terrestrial and bacterial FAB (p = 0.05, p = 0.15, respectively). Contrary to the other species, *Daphnia* showed little phytoplankton FAB accumulation over the year (1.85 µg mg DW^−1^) and no significant difference between ice-covered and ice-free season in phytoplankton (log transformed; t = −1.25, p = 0.26), terrestrial or bacterial FAB (p > 0.50).

**Figure 5:**
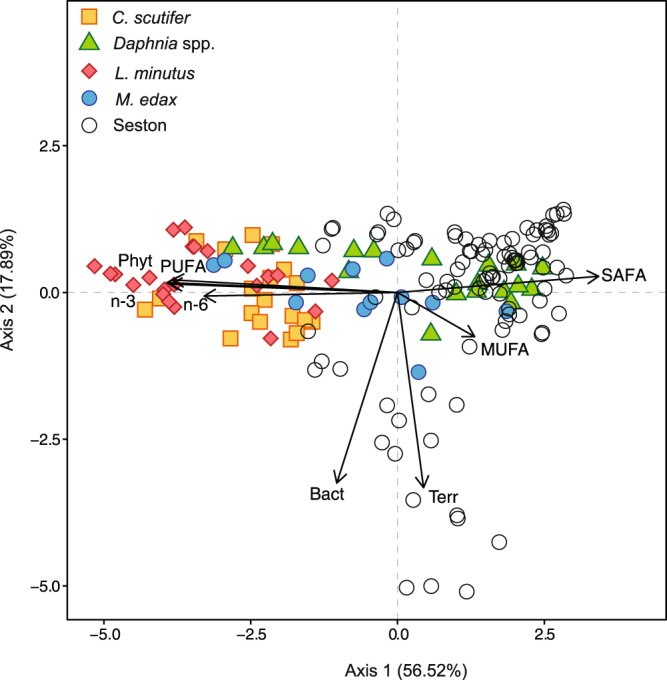
Principal component analysis (PCA) on FA composition grouped as polyunsaturated fatty acids (PUFA), monounsaturated fatty acids (MUFA), saturated fatty acids (SAFA), biomarkers of phytoplankton (Phyt), terrestrial (Terr), bacteria (Bact), omega-3 fatty acids (n-3) and omega-6 fatty acids (n-6). Proportion of explained variance per axes is in parentheses.

Principal component analysis captured 74% of the total variation in zooplankton FA composition, with 57% to axis 1 and 18% to axis 2 (Fig. 5). PUFA, phytoplankton FAB, n-3 FA and SAFA contributed 22%, 21%, 21% and 17%, respectively, to axis 1. Terrestrial FAB and bacterial FAB contributed 49% and 47%, respectively, to axis 2. *L. minutus* and *C. scutifer* were associated with axis 1 corresponding to high PUFA and phytoplankton FAB. The seston samples were characterised mainly by the presence of SAFA as were also the majority of *Daphnia* samples. SAFA composition in seston and *Daphnia* were dominated by C16:0 and C18:0 (see Supplementary Table S3). Some of the seston samples were further associated with bacterial and terrestrial FA. The composition of FA among taxonomic groups (*L. minutus*, *C. scutifer*, *M*. edax, *Daphnia* spp. and seston) was significantly different (PERMANOVA, F_(4,122)_ = 93.2, p = 0.001).

## Discussion

Our results demonstrate that the usually overlooked winter season involves key ecosystem processes. The high availability of phytoplankton PUFA in seston and their efficient accumulation by copepods establish a critical link between primary producers and higher trophic levels. By use of fatty acid biomarkers (FAB), we showed that during times of low primary production in winter, contrary to our hypotheses, the prevalence of terrestrial organic matter and/or bacteria did not increase in copepod or cladoceran lipid reserves. Instead, early winter accumulation of phytoplankton-derived PUFA and their progressive decline in copepod lipid reserves in mid- and late-winter suggests that phytoplankton FA are critical for several species of zooplankton to survive and remain in an active stage until spring.

Energy to sustain winter metabolism and survival can be obtained from lipid reserve accumulation that has been considered to take place in autumn before the lake freezes^12^. Our experiment on *L. minutus* demonstrated that also early winter is a critical accumulation period; the individuals had not accumulated enough FA at the moment of ice formation to be able to survive the entire winter without external food input. At the ice-on, when the experiment started and the *L. minutus* were collected from the lake, the copepods had accumulated lipid reserves to survive for two months in laboratory, which carried them without additional food to February. However, the seston FA composition in the lake showed that the phytoplanktonic FA were available until January, i.e. for about one month after the ice had formed (Fig. 3). If we extrapolate the survival time without food, obtained from the experiment (66 days), we can calculate that in order to survive until the ice melting (18 April), *L. minutus* could have stopped eating on 12 February. This corresponds well with the moment the lake population of copepods ceased to accumulate FA reserves (19 January, Fig. 4), indicating FA in seston were exhausted. Interestingly, they started to use the FA reserves only a month later (29 February), probably corresponding to a minimum metabolic activity in mid-winter. Lake Simoncouche phytoplankton production begins before the ice disappears^4^ providing fresh food for the overwintering populations in late winter. We argue that had the *L. minutus* remained in the lake longer before the initiation of the experiment they would have been able to accumulate the reserves needed to carry them over the months when there was no food in the water column. Few studies have estimated the survival of similar species under starved conditions. Elendt and Storch^38^ discussed that *L. minutus* can survive 15.4 days, *M. edax* 24.3 days and *Daphnia magna* only 7.6 days. However, these authors do not report season, lipid content or temperature at which these survival times were estimated, thus limiting a direct comparison with our experiment. Nevertheless, the shorter survival time of *Daphnia* under starved conditions is in accordance with our abundance data that showed that *Daphnia* abundance declined close to zero once the seston PUFA pool was exhausted (Figs 1, 3).

The concentration of phytoplankton FA in seston peaked just before the ice-in and just after the ice-out, and followed the seasonal pattern of phytoplankton FA production in Lake Simoncouche^36^. The autumn peak decreased gradually but remained at a relatively high level for about a month under the ice (Fig. 3). Historically, phytoplankton primary production, like every biological activity, is assumed negligible in winter, but several recent studies reported under-ice primary production in oceans^39^ and in many lakes^40^. A seasonal pattern similar to our seston FA composition has also been shown for lakes in the northern USA, with a very high spring bloom associated with phytoplankton FA abundance and a relatively high abundance in autumn^41^. Usually eicosapentaenoic acid (EPA, 20:5n-3) and docosahexaenoic acid (DHA, 22:6n-3) content in phytoplankton are limited by nitrogen and phosphorous concentrations in water^42, 43^. Summer TDN and TDP shortage in Lake Simoncouche (Table 1) was followed by a nutrient increase during lake mixing (October). It appears plausible that the autumn nutrient increase in the water column allows the phytoplankton community to synthesize new PUFA that the nutrient depletion in summer does not permit. Also, n-3 FA are synthesized by different phytoplankton species^29^, and vertical mixing has been shown to cause major changes in phytoplankton community composition^44^. These phenomena may explain the n-3 FA maximum in autumn.

All FAB increased in quantity when the ice formed, and then decreased to their annual minimum by mid-winter (Fig. 3). The winter terrestrial FA minimum coincided with the minimal values of SUVA, indicating decreased input of terrestrial matter from the frozen catchment soils^45^. The minimum concentration of bacterial FA coincided with the minimum in bacterial biomass in winter. The same observation was valid for the autumnal FA peak confirming that seasonal changes in terrestrial and bacterial FAB concentrations followed the presence of terrestrial or bacterial material in seston.

The high variability in FA content and composition among species indicate different adaptations to winter and different life strategies^12^ with species that stay active in winter having higher lipid content and FA composition dominated by PUFA in autumn (Fig. 5). Earlier studies have assumed that reserve accumulation would take place in autumn before ice formation^12^ and have suggested that zooplankton strongly reduce or even cease their food intake under the ice^17^. Here, the copepods *L. minutus* and *C. scutifer* showed a strong ability to accumulate lipids until January, up to 76% of the biomass (see Supplementary Fig. S1), emphasizing the importance of early winter in plankton ecology, a season that has been largely ignored in limnological studies. Yet, this lipid accumulation and over-wintering strategies have already been observed in herbivorous calanoids from marine polar environment^19^. We propose that the autumnal and early winter lipid accumulation allows certain zooplankton to spend the entire winter in an active form either as adults (*L. minutus*) or as C-IV stages (*C. scutifer*), which are the two strategies observed for these species^4, 46^. As lack of food and cold temperatures are considered stress factors, *Daphnia* are usually believed to cope with this stress by producing resting eggs (ephippia; Hiruta and Tochinai^47^). Our observations indicate that the majority of *Daphnia* in Lake Simoncouche followed this strategy (Fig. 1), however the individuals that overwintered actively did seemingly well even in late January when they were observed to carry parthenogenetic young (Grosbois, unpublished data, see Supplementary Fig. S3). The cyclopoid *M. edax* also disappeared from the water column to the sediments from November to May as C-V stage copepodites, possibly as a strategy to avoid predators or because they do not have the physiological ability to accumulate lipids.

When consumed, the branched FA common to bacteria are transferred to higher trophic levels without modification and can be measured in the copepod FA composition^48^. The branched and saturated FA have been shown to be associated to growth and membrane fluidity regulation in bacteria^49^ but their physiological role in zooplankton is less known. Because of the lack of n-3 and n-6 PUFA, bacteria are commonly considered as a poor food source with negative effects on zooplankton growth and reproduction^50^. Similarly, the FA quality of terrestrial material is considered to be poorer than that of aquatic material^30^. However, terrestrial organic matter is known to be selectively assimilated in bacteria biomass^51^ and then probably transferred to higher trophic levels via heterotrophic eukaryotic protists and rotifers, therefore making it available to crustacean zooplankton. During this transfer the nutritional quality of terrestrial organic material and bacteria can be trophically upgraded by heterotrophic flagellates^52^ and ciliates^53^. Our results, however, show that despite the potential trophic upgrade of terrestrial and bacterial material, terrestrial and bacterial FAB were not substantially accumulated by zooplankton at any time of the year. The maximal contribution of terrestrial (2.1%) and bacterial FAB (2.9%) in zooplankton was measured for *C. scutifer* in December and February showing that these biomarkers played a small role in the total FA accumulation in the four studied zooplankton species. Our results therefore do not give support to earlier literature that has suggested that zooplankton switch to terrestrial organic carbon sources in lack of phytoplankton production^54, 55^. It is important to note that our study does not exclude the possibility that zooplankton use terrestrial organic matter for respiration and/or to support cell and tissue renewal. However, the results confirm that terrestrial and bacterial molecules are not preferentially stored in zooplankton lipid reserves, even when terrestrial and bacterial FAB are present and available to zooplankton in seston. This observation is in accordance with the results of Mariash, *et al*.^56^, who showed an absence of SAFA trophic accumulation indicating a lack of terrestrial and bacterial FA accumulation in cladoceran lipid reserves.

Among all the FA accumulated by copepods in winter, PUFA from phytoplankton were the most accumulated demonstrating the predominant role of these FA in the reserve accumulation of organisms that stay active under the ice (Figs 4, 5). These FA are important regulators of membrane fluidity, which is reduced at low temperatures^57^, and are used by the organisms to cope with winter conditions. They have also been suggested to contribute to organism’s metabolic maintenance as well as to investment of n-3 FA into reproduction in late winter and early spring^4^. Both *L. minutus* and *C. scutifer* accumulated these FA, especially DHA (see Supplementary Table S3), which is an essential FA for copepod reproduction^58^. As *C. scutifer* needed to mature from C-IV stage to adult in winter and then reproduce (Fig. 1), it is very likely that the accumulated PUFA were used primarily for growth and then reproduction. FA composition differed among taxa and characterized winter abundant species (*L. minutus* and *C. scutifer* copepods) with high dominance of PUFA from phytoplankton in their accumulated lipids and winter low abundant or absent species (*Daphnia* spp. and *M. edax*) with SAFA dominance (Fig. 5). None of these species accumulated bacterial or terrestrial FA substantially. SAFA dominance in *Daphnia* and *M. edax* were characterized by the generalist C16:0 and C18:0 FA rather than terrestrial LC-SAFA or bacterial Br-SAFA (Supplementary Table S3). Ephippia-producing *Daphnia* that disappear from the water column in winter do not accumulate FA to biomass but rather invest them to resting eggs (ephippia), in particular EPA^59^ that was largely available in seston before winter. The concentration of phytoplankton FA in *Daphnia* started to increase in April indicating that phytoplankton FA were produced in the seston and available for consumers before the ice melted. *M. edax* predation on *Daphnia* (see Supplementary Fig S2) was confirmed by their similar FA composition (Fig. 5). Collectively, these results demonstrate that the use and accumulation of FA is closely related to the species life strategy.

To conclude, this study demonstrates that the availability of phytoplankton FA remained high in seston in early winter permitting copepods to accumulate and subsequently metabolise n-3 FA throughout winter, thereby providing a mechanism for under ice growth and reproduction^4^. Although the mechanism is known for some marine organisms especially in polar ecosystems, no information has earlier been available for freshwaters. To our knowledge, our study also reports for the first time seston and zooplankton bacterial and terrestrial FAB for a complete year. These terrestrial and bacterial FAB were not accumulated by zooplankton in winter, suggesting that terrestrial organic material and bacteria are not used as alternative resources during times of low primary production under the ice. The duration of ice cover is predicted to be shorter in the future with climate change^60^, phytoplankton with low PUFA content such as cyanobacteria are predicted to increase in the next decades^61^, and the worldwide FA source is predicted not to meet future human needs^62^. It is therefore essential to better understand how PUFA accumulation by zooplankton is seasonally regulated, how it will be affected by changing ice conditions, and how these changes will drive modifications in FA composition of long-lived consumers such as fish.

## Methods

### Study site and zooplankton community

We sampled Lake Simoncouche (48°23′N, 71°25′W), a medium size mesotrophic shallow lake (mean depth 2.2 m, maximum depth 8 m and surface area 83 ha) situated in Quebec, Canada for one complete year. The lake shows a typical boreal seasonality with ice forming in November and melting in late April to early May. The maximum ice thickness is 50–90 cm (February), capped with about 40 cm of snow. The catchment basin spreads on 2,543 ha and is covered by a boreal forest dominated by *Abies balsamea*, *Picea mariana* and *Betula papyrifera* ^63^. Zooplankton crustacean community is dominated by *Leptodiaptomus minutus* (Lilljeborg 1889), *Cyclops scutifer* (Sars 1863), *Mesocyclops edax* (S.A. Forbes 1891) and *Daphnia* spp. with occasional presences of *Epishura lacustris* (S.A. Forbes 1882), *Aglaodiaptomus spatulocrenatus* (Pearse, 1906), *Mesocyclops leuckarti* (Claus, 1857), *Tropocyclops prasinus* (Fischer, 1860), *Eucyclops speratus* (Lilljeborg, 1901) as well as the cladocerans *Bosmina* spp., *Diaphanosoma* spp., *Holopedium* sp. (Zaddach, 1855). Invertebrate predators in the pelagic environment are represented by *Leptodora kindtii* (Focke, 1844) and *Chaoborus* sp.

### Survival experiment

We designed a factorial laboratory experiment to estimate zooplankton survival in fed versus starved conditions over time. The experiment lasted 164 days; it started when ice was forming on Lake Simoncouche (20 November 2012) and ended five months later when the ice disappeared (3 May 2013). The experiment was run with the calanoid copepod *Leptodiaptomus minutus*, a common species in eastern North-America that dominates many boreal lakes^64^. In Lake Simoncouche it makes up to 93% of the total zooplankton biomass (Grosbois unpublished). The copepods were sampled from the pelagic zone of the lake via vertical net tows and transferred to 1 L plastic containers with lake water and brought to the laboratory. Both treatment modalities were composed of six replicates (N_tot_ = 12) of fifty copepods each, which were selected under a stereomicroscope (Discovery V12, Zeiss, Jena, Germany, ×8-x100) and transferred to 250 mL beakers using a pipette. Half of the beakers (6) contained GF/F-filtered lake water and were considered the starved treatment, the other 6 beakers had lake water (<50 µm) providing the copepods with a natural food supply present in the lake (control). The beakers were installed at 4 °C in the dark to simulate lake conditions in winter. Water was entirely renewed weekly with fresh Lake Simoncouche water that was either sieved (50 µm) or GF/F-filtered depending on the treatment, and the copepods were counted, survival noted, and dead individuals removed.

### Water and zooplankton sampling

Lake water was collected monthly from 12 January 2011 to 16 May 2012 for total dissolved nitrogen (TDN), total dissolved phosphorous (TDP), dissolved organic carbon (DOC), specific UV-absorbance (SUVA_254_), chlorophyll *a* (Chl-a), bacterial biomass (Bact Biom) and from 6 July 2011 to 7 May 2013 for fatty acid (FA) analysis. Samples were taken from the epilimnion at the deepest point of the lake using a 2 L Limnos sampler (Limnos Ltd, Turku, Finland). Water was collected from every meter, pooled to one integrated sample in a bucket and stored in a 4 L Nalgene container. Water for FA analysis was collected from the metalimnion as well (hypolimnion does not form in this shallow lake). In the laboratory, subsamples of water for TDN, TDP, DOC and SUVA were filtered through a cellulose acetate filter (0.2 µm) that had been pre-rinsed with Milli-Q water. TDN, TDP, and DOC were analysed using a Lachat Autoanalyser, a ThermoSpectronic spectrophotometer and a Shimadzu TOC-V, respectively, at the Institut national de la recherche scientifique (INRS), Quebec City, Canada. SUVA_254_, an index of DOC aromaticity associated with allochthonous (terrestrial) carbon sources^45^, was measured as absorbance at 254 nm using a Cary 100 UV-Vis spectrophotometer (Agilent, Santa Clara, U.S.A.) and normalized to DOC concentration. Samples for Chl-a (three replicates on each date) were filtered onto GF/F filters that were then folded, wrapped in aluminium foil and stored at −80 °C until spectrofluorometric analysis as in Mush^65^ using a Cary Eclipse fluorescence spectrophotometer (Agilent, Santa Clara, USA). Aliquots for bacterial biomass were first preserved with formaldehyde (2% final concentration), then stained with 4.6-diamido-2-phenylindole (DAPI, final concentration 5 µg ml^−1^) and filtered onto black cellulose filters (0.2 µm) that were mounted onto microscope slides (three replicates for each date) and stored at −20 °C until bacteria cell counting using epifluorescence microscopy with a UV excitation (365 nm) filter and an inverted microscope (Axio Observer A1, Zeiss, Jena, Germany × 1000). Seston samples for FA (three replicates for each date) were filtered onto pre-combusted and pre-weighted GF/F filters that were then folded, wrapped in aluminium foil and stored at −80 °C until freeze-drying.

Zooplankton was sampled weekly to monthly from 19-May-2011 to 23-May-2012 for identification, abundance, and FA. The samples for FA were collected by vertical net tows with a 50 µm mesh size net (diameter 25 cm) from the entire water column (0–6 m). To estimate zooplankton abundance, water samples (6 to 20 L) were collected weekly with a Limnos sampler from several depths and concentrated to one integrated sample using a 50 µm sieve. Formaldehyde (final concentration: 4%) was added to the abundance samples until identification^66, 67^ with an inverted microscope (Axio Observer A1, Zeiss, Jena, Germany, ×100) and Utermöhl chambers. Copepod identification was carried out for copepodites and adults further divided into males and females. Cladocerans were counted without considering developmental stages. When zooplankton density was too high, half or a quarter of the sample was counted after division in Folsom’s sample divider. A minimum of 100 individuals were identified per sample except for 7 low density samples (N_tot_ = 71) where about 60 individuals were counted. For zooplankton FA analyses, 100–200 individuals were carefully collected with forceps under stereomicroscope, placed in Eppendorf tubes (1.5 mL) and stored at −80 °C until freeze-drying. Fatty acids were analysed from the 4 most abundant taxa of the community: *Leptodiaptomus minutus*, *Cyclops scutifer*, *Mesocyclops edax* and *Daphnia* spp.

### Fatty acid analyses

Lipids were extracted from freeze dried seston and zooplankton in chloroform-methanol mixture following Heissenberger, *et al*.^68^. Lipid concentration in zooplankton was calculated from lipid mass measured by gravimetry and from zooplankton mass obtained using a micro-balance (XP26 DeltaRange, Mettler Toledo, Greifensee, Switzerland). As such extraction also includes molecules such as lipid head groups, sterols and carotenoids we further quantified only the FA in the samples. Toluene was added to lipid extracts and each sample was trans-esterified at 50 °C during 15 hours with 1% methanolic sulfuric acid. The resulting fatty acid methyl esters (FAME) were separated from the rest of the material by adding KHCO_3_ − water (2% v/v) and hexane. FAME were then identified and quantified by gas chromatography-mass spectrometry (GC-MS) using an Agilent 7890 A chromatograph (Agilent Technologies, Santa Clara, CA) equipped with an Agilent 5975 C mass spectrometer with triple-axis detector and an Agilent J&W DB-23 column (60 m length, 0.25 mm inner diameter, 0.15 µm film thickness). Samples were injected in splitless mode, temperature ramp was 70 °C for 1.5 min with different steps of 20 °C per minute until 110 °C, 12.5 °C per minute until 160 °C and 2.5 °C per minute until 230 °C with a hold time of 6.5 minutes. The gas flow was 1.0032 ml per minute. The instrument was run in Single Ion Monitoring mode to ensure lowest possible limits of detection and most precise quantification. The resulting retention time and ion composition were used for FAME identification using different FAME and bacterial acid methyl esters mix standards (Sigma-Aldrich), and the compound identity was confirmed with the limited mass spectrums of the standards. The peak area of the most abundant FA specific ion (m/z 74, 79, 81 and 87) versus an internal standard (nonadecanoic acid, Sigma-Aldrich, N5252) was used for FAME quantification using calibration curves based on known standard concentrations.

Biomarkers documented only for a unique taxon (phytoplankton, terrestrial plant or bacteria) were selected from freshwater, marine and terrestrial literature (see Supplementary Table S1). The unsaturated FA C18:4n-3, C20:1n-9, C20:4n-6, C20:5n-3, C22:6n-3, C24:1n-9 were selected as phytoplankton biomarkers, the long-chained saturated FA C20:0, C22:0, C23:0, C24:0 were selected as terrestrial biomarkers and the branched-chained saturated FA aC15:0, iC15:0, iC17:0 as well as the saturated FA (SAFA) C15:0 and the Cyclic SAFA Cy-C17:0 were selected as bacterial biomarkers.

### Statistical analyses

Treatments from the survival experiment were tested with repeated measures ANOVA and refined with contrasts for each date using a non-parametric Wilcoxon test, as the data did not meet the assumptions of homoscedasticity and normality. Kolmogorov-Smirnov and Bartlett’s tests were used to test data normality and homoscedasticity respectively. Alpha level was set at 0.05. Principal component analyses (PCA) were applied to normalized FA composition data that was grouped according to FA biomarkers (FAB), polyunsaturated fatty acids (PUFA), n-3 FA, n-6 FA, monounsaturated fatty acids (MUFA) and saturated fatty acids (SAFA). Differences of FA composition among taxa were tested with PERMANOVA in PRIMER v.6.1.11 & PERMANOVA + v.1.0.1^69^. FAB concentrations were compared using student’s t-test. All analyses except PERMANOVA were carried out in the R environment v.3.3.1^70^.

## Data Availability

Most of the data generated or analysed during this study are included in this published article (and its Supplementary Information files). Other datasets are available from the corresponding author on reasonable request.

## Electronic supplementary material

Supplementary Material
