# Supplementary material for: Under-ice availability of phytoplankton lipids is key to freshwater zooplankton winter survival
Source: Sci Rep. 2017 Sep 14;7:11543. doi: 10.1038/s41598-017-10956-0 (PMC5599675; doi:10.1038/s41598-017-10956-0)
Supplement: Supplementary file 1 — Supplementary Material [file 41598_2017_10956_MOESM1_ESM.pdf]

Under-ice availability of phytoplankton lipids is key to zooplankton winter survival

Guillaume Grosbois*[1], Heather Mariash[1,2], Tobias Schneider[1] and Milla Rautio[1]

Supplementary material

Table

Table S1

Selected fatty acid biomarkers for phytoplankton, terrestrial organic matter and bacteria.

Fatty acid	Abbrev-iation	Biomarker	Reference
C18:4n-3	SDA	Phytoplankton	Cook et al. (2000), Hughes et al. (2005), Ackman (1989), Sleigh (1987), Mai et al. (1996), McLeod and Wing (2009), Taipale et al. (2013), Taipale et al. (2015b), Stevens et al. (2004).
C20:1n-9		Phytoplankton	Taipale et al. (2015b)
C20:4n-6	ARA	Phytoplankton	Paradis and Ackman (1977), Cook et al. (2000), Isay and Busarova (1984), Mai et al. (1996), Nelson et al. (2002), Hughes et al. (2005), Kharlamenko et al. (1995), McLeod and Wing (2009), Taipale et al. (2015b)
C20:5n-3	EPA	Phytoplankton	Berggren et al. (2014), Ackman et al. (1968), Paradis and Ackman (1977), Isay and Busarova (1984), Ackman (1989), Mai et al. (1996), Cook et al. (2000), Nelson et al. (2002), Hughes et al. (2005), Howell et al. (2003), Boschker et al. (2005), Ratledge and Wilkinson (1988), Dunstan et al. (1993), McLeod and Wing (2009), Kharlamenko et al. (1995), Taipale et al. (2015b)
C22:6n-3	DHA	Phytoplankton	Howell et al. (2003), Sleigh (1987), Hamilton (1995), Vazhappilly and Chen (1998), Mansour et al. (1999), Hughes et al. (2005), McLeod and Wing (2009),

		Simopoulos (1991), Stevens et al. (2004), McMeans et al. (2015)
C24:1n-9	Phytoplankton	Taipale et al. (2015b)
C20:0	Terrestrial	Taipale et al. (2015b)
C22:0	Terrestrial	Taipale et al. (2015b), Wenzel et al. (2012), McMeans et al. (2015)
C23:0	Terrestrial	Taipale et al. (2015b)
C24:0	Terrestrial	Taipale et al. (2015b), Zelles (1999), Ruess et al. (2007), Bachok et al. (2003), McLeod and Wing (2009), McMeans et al. (2015)
a-C15:0	Bacteria	Haubert et al. (2006), McLeod and Wing (2009), Taipale et al. (2015b), Pancost and Sinninghe Damsté (2003), McMeans et al. (2015)
C15:0	Bacteria	Taipale et al. (2015b), McMeans et al. (2015)
Cy-C17:0	Bacteria	Haubert et al. (2006), (Zelles 1997, 1999)
i-C15:0	Bacteria	Haubert et al. (2006), McLeod and Wing (2009), Taipale et al. (2015b), McMeans et al. (2015)
i-C17:0	Bacteria	Haubert et al. (2006), Pancost and Sinninghe Damsté (2003), McMeans et al. (2015)

Table S2

Paired Wilcoxon tests between “Control” and “Starved” treatments per date. p = NA when each replicates had 50 individuals at the beginning of the experiment and no test could be completed.

#	Date	Duration (d)	W	p	N
1	24/11/2012	0	18	NA	12
2	30/11/2012	6	18	NA	12

3	04/12/2012	10	16	0.806	12
4	11/12/2012	17	24	0.372	12
5	18/12/2012	24	23	0.462	12
6	28/12/2012	34	16	0.808	12
7	03/01/2013	40	23	0.462	12
8	08/01/2013	45	23	0.462	12
9	15/01/2013	52	23	0.462	12
10	22/01/2013	59	23	0.462	12
11	29/01/2013	66	28	0.128	12
12	05/02/2013	73	36	0.005 **	12
13	12/02/2013	80	36	0.005 **	12
14	19/02/2013	87	36	0.004 **	12
15	27/02/2013	94	36	0.004 **	12
16	05/03/2013	101	36	0.004 **	12
17	12/03/2013	108	36	0.004 **	12
18	19/03/2013	115	36	0.004 **	12
19	26/03/2013	122	36	0.004 **	12
20	02/04/2013	129	36	0.004 **	12
21	10/04/2013	137	36	0.004 **	12
22	16/04/2013	143	36	0.004 **	12
23	23/04/2013	150	36	0.004 **	12
24	29/04/2013	156	36	0.004 **	12
25	05/05/2013	162	36	0.004 **	12

Table S3

Raw individual FA (μg FA mg dry weight^{-1}) data

Date	Month	Material	Species	C14:0	C14:1n-5	C15:0	C15:1n-5	C16:0	C16:1n-7	C17:0	c17:1n-7	C18:0	C18:1n-7	C18:1n-9	C18:2n-6	C18:3n-3	c18:3n-6	C18:4n-3	C20:0	C20:1n-9	C20:1n-11	C20:2n-6	C20:3n-3	C20:3n-6	C20:4n-6	C20:5n-3	C21:0	C22:0	C22:1n-9	C22:2n-6	C22:6n-3	C23:0	C24:0	C24:1n-9	i-C15:0	a-C15:0	i-C16:0	i-C17:0	C17:0^	2-OH C14:0	3-OH C14:0	2-OH C16:0
19/05/2011	May	Zooplankton	*Cyclops scutifer*	0.84	0.00	0.12	0.00	4.19	0.03	0.00	0.03	1.43	0.00	2.05	0.91	1.05	0.23	1.02	0.06	0.00	0.09	0.06	0.12	0.03	0.18	1.55	0.00	0.03	0.03	0.00	3.35	0.00	0.03	0.15	0.09	0.03	0.00	0.06	0.00	0.03	0.00	0.00
19/05/2011	May	Zooplankton	*Cyclops scutifer*	1.02	0.00	0.12	0.00	4.07	0.07	0.17	0.02	1.25	0.02	1.73	0.79	0.87	0.19	0.79	0.05	0.02	0.07	0.05	0.07	0.02	0.24	1.40	0.00	0.02	0.02	0.00	2.76	0.00	0.02	0.10	0.10	0.05	0.00	0.07	0.00	0.02	0.00	0.00
22/06/2011	June	Zooplankton	*Cyclops scutifer*	2.90	0.03	0.41	0.02	6.97	2.85	0.31	0.07	2.49	0.04	3.08	2.24	2.28	0.01	1.60	0.20	0.02	0.18	0.27	0.39	0.12	1.21	3.03	0.03	0.14	0.04	0.02	4.44	0.02	0.04	0.18	0.29	0.10	0.01	0.15	0.00	0.05	0.01	0.00
22/06/2011	June	Zooplankton	*Cyclops scutifer*	1.87	0.01	0.28	0.01	5.00	1.96	0.23	0.04	1.77	0.02	1.92	1.37	1.54	0.00	1.05	0.11	0.01	0.11	0.15	0.23	0.07	0.69	1.93	0.01	0.09	0.02	0.01	3.01	0.01	0.03	0.13	0.18	0.06	0.00	0.10	0.00	0.03	0.01	0.00
13/07/2011	July	Zooplankton	*Cyclops scutifer*	3.27	0.02	0.68	0.03	8.82	4.53	0.55	0.14	3.03	0.06	4.60	2.98	3.77	0.01	2.24	0.35	0.04	0.39	0.51	0.84	0.25	1.89	4.28	0.05	0.26	0.08	0.03	5.44	0.03	0.09	0.43	0.46	0.15	0.01	0.25	0.00	0.07	0.02	0.00
13/07/2011	July	Zooplankton	*Cyclops scutifer*	2.31	0.02	0.57	0.02	8.46	4.07	0.54	0.13	3.19	0.05	4.70	3.21	3.78	0.01	2.23	0.33	0.03	0.36	0.46	0.78	0.23	1.80	4.25	0.04	0.23	0.07	0.03	5.54	0.03	0.09	0.40	0.36	0.12	0.01	0.23	0.00	0.06	0.02	0.00
17/08/2011	August	Zooplankton	*Cyclops scutifer*	1.71	0.01	0.53	0.02	6.82	3.65	0.58	0.12	2.38	0.04	3.41	1.99	2.65	0.01	1.15	0.29	0.03	0.29	0.33	0.49	0.16	1.27	2.32	0.04	0.21	0.06	0.02	3.76	0.03	0.09	0.39	0.31	0.11	0.01	0.21	0.00	0.04	0.02	0.00
17/08/2011	August	Zooplankton	*Cyclops scutifer*	3.12	0.03	0.75	0.03	8.10	4.66	0.62	0.15	2.54	0.05	3.76	2.18	2.94	0.01	1.34	0.31	0.03	0.27	0.36	0.57	0.19	1.36	2.54	0.04	0.22	0.07	0.02	3.73	0.03	0.09	0.42	0.49	0.16	0.01	0.23	0.00	0.05	0.02	0.00
26/10/2011	October	Zooplankton	*Cyclops scutifer*	1.92	0.00	0.21	0.00	4.79	2.49	0.00	0.03	1.51	0.00	1.05	0.81	0.97	0.27	1.59	0.08	0.00	0.05	0.08	0.11	0.08	0.35	1.62	0.03	0.08	0.03	0.00	2.79	0.00	0.05	0.14	0.24	0.05	0.00	0.11	0.00	0.00	0.00	0.00
12/12/2011	December	Zooplankton	*Cyclops scutifer*	0.46	0.01	0.04	0.00	2.44	1.53	0.00	0.02	1.44	0.02	3.50	1.52	1.35	0.27	3.16	0.20	0.04	0.28	0.22	0.28	0.36	0.93	4.03	0.06	0.34	0.08	0.02	7.36	0.03	0.27	0.59	0.05	0.02	0.04	0.14	0.00	0.02	0.01	0.00
12/12/2011	December	Zooplankton	*Cyclops scutifer*	4.58	0.04	0.23	0.01	5.61	4.77	0.01	0.05	1.55	0.02	4.03	1.86	1.74	0.35	4.16	0.17	0.03	0.24	0.20	0.24	0.31	0.91	3.86	0.05	0.25	0.06	0.02	6.18	0.02	0.20	0.45	0.42	0.12	0.12	0.24	0.00	0.02	0.01	0.00
19/01/2012	January	Zooplankton	*Cyclops scutifer*	0.22	0.01	0.06	0.01	3.57	2.18	0.00	0.03	1.70	0.01	4.20	1.85	1.83	0.29	3.55	0.21	0.04	0.32	0.28	0.35	0.34	1.03	4.36	0.06	0.31	0.10	0.03	7.56	0.03	0.23	0.60	0.07	0.03	0.06	0.20	0.00	0.03	0.01	0.00
19/01/2012	January	Zooplankton	*Cyclops scutifer*	7.44	0.04	0.39	0.03	8.01	6.64	0.01	0.08	2.14	0.04	5.28	2.65	2.68	0.43	5.35	0.24	0.05	0.35	0.33	0.41	0.40	1.25	5.07	0.07	0.30	0.11	0.03	8.36	0.03	0.24	0.61	0.76	0.22	0.20	0.37	0.00	0.04	0.03	0.00
29/02/2012	February	Zooplankton	*Cyclops scutifer*	9.32	0.07	0.49	0.04	7.75	7.12	0.01	0.10	2.00	0.06	5.53	2.54	2.41	0.39	4.51	0.23	0.05	0.32	0.33	0.36	0.38	1.24	4.33	0.06	0.31	0.11	0.03	6.99	0.03	0.22	0.64	0.93	0.27	0.22	0.37	0.00	0.05	0.03	0.00
29/02/2012	February	Zooplankton	*Cyclops scutifer*	5.71	0.02	0.41	0.02	8.08	0.37	0.01	0.08	2.48	0.05	6.14	2.70	2.46	0.40	4.52	0.26	0.06	0.38	0.35	0.40	0.42	1.37	4.78	0.07	0.35	0.12	0.04	8.31	0.04	0.30	0.83	0.66	0.21	0.20	0.40	0.00	0.05	0.02	0.00
04/04/2012	April	Zooplankton	*Cyclops scutifer*	5.86	0.01	0.49	0.06	6.89	6.21	0.00	0.15	1.80	0.02	4.18	1.94	2.10	0.27	3.04	0.16	0.03	0.23	0.22	0.24	0.22	0.81	3.25	0.03	0.20	0.08	0.02	5.02	0.02	0.14	0.49	0.64	0.23	0.00	0.28	0.00	0.03	0.02	0.00
04/04/2012	April	Zooplankton	*Cyclops scutifer*	6.14	0.02	0.49	0.05	7.00	6.30	0.00	0.14	1.73	0.04	4.16	2.05	2.26	0.28	3.24	0.17	0.03	0.23	0.24	0.25	0.24	0.88	3.47	0.04	0.20	0.08	0.02	5.27	0.02	0.14	0.52	0.69	0.24	0.18	0.29	0.00	0.03	0.02	0.00
23/05/2012	May	Zooplankton	*Cyclops scutifer*	2.15	0.01	0.18	0.00	4.52	1.85	0.00	0.02	1.27	0.02	1.21	1.19	1.12	0.00	1.13	0.07	0.00	0.08	0.10	0.13	0.06	0.79	2.35	0.01	0.07	0.03	0.01	5.30	0.01	0.04	0.18	0.16	0.08	0.00	0.07	0.00	0.02	0.00	0.00
23/05/2012	May	Zooplankton	*Cyclops scutifer*	1.24	0.00	0.15	0.01	3.89	1.58	0.00	0.04	1.33	0.02	1.11	1.13	1.02	0.00	0.97	0.07	0.00	0.08	0.10	0.12	0.06	0.83	2.38	0.01	0.07	0.02	0.01	5.39	0.01	0.05	0.18	0.10	0.05	0.00	0.06	0.00	0.02	0.00	0.00
19/05/2011	May	Zooplankton	*Daphnia*	1.45	0.02	0.13	0.00	5.07	0.51	0.00	0.07	1.40	0.00	3.88	1.35	2.30	0.38	3.36	0.02	0.00	0.00	0.02	0.00	0.02	0.80	3.93	0.00	0.02	0.00	0.00	0.22	0.00	0.00	0.00	0.15	0.09	0.00	0.09	0.11	0.02	0.00	0.00
22/06/2011	June	Zooplankton	*Daphnia*	0.49	0.01	0.12	0.00	3.25	0.06	0.13	0.02	0.90	0.00	2.71	0.28	0.13	0.09	0.00	0.01	0.00	0.00	0.00	0.00	0.00	0.05	0.04	0.00	0.01	0.00	0.00	0.00	0.00	0.00	0.00	0.09	0.02	0.00	0.00	0.00	0.00	0.00	0.00
22/06/2011	June	Zooplankton	*Daphnia*	0.63	0.01	0.17	0.01	3.48	1.92	0.00	0.02	1.40	0.00	3.08	1.37	0.76	0.00	0.38	0.05	0.00	0.00	0.01	0.01	0.01	0.64	0.97	0.00	0.02	0.00	0.00	0.04	0.00	0.01	0.00	0.00	0.04	0.00	0.06	0.00	0.01	0.00	0.00
13/07/2011	July	Zooplankton	*Daphnia*	0.49	0.00	0.14	0.02	3.08	0.12	0.18	0.04	1.07	0.00	2.36	0.46	0.32	0.14	0.09	0.02	0.00	0.00	0.00	0.00	0.00	0.14	0.14	0.00	0.02	0.00	0.00	0.00	0.00	0.00	0.00	0.09	0.03	0.02	0.07	0.00	0.00	0.00	0.00
13/07/2011	July	Zooplankton	*Daphnia*	0.60	0.00	0.18	0.01	3.67	0.18	0.22	0.04	1.45	0.00	2.80	0.83	0.96	0.00	0.43	0.03	0.00	0.00	0.01	0.01	0.01	0.65	1.11	0.00	0.03	0.00	0.00	0.04	0.00	0.01	0.00	0.12	0.04	0.00	0.07	0.00	0.01	0.00	0.00
17/08/2011	August	Zooplankton	*Daphnia*	0.79	0.02	0.23	0.00	4.23	3.05	0.28	0.04	1.20	0.00	3.09	0.58	0.58	0.00	0.15	0.02	0.00	0.00	0.00	0.00	0.00	0.24	0.24	0.00	0.02	0.00	0.00	0.00	0.00	0.00	0.00	0.15	0.04	0.00	0.06	0.00	0.00	0.00	0.00
17/08/2011	August	Zooplankton	*Daphnia*	0.15	0.00	0.13	0.00	3.07	0.13	0.23	0.05	1.10	0.00	2.60	0.25	0.15	0.18	0.00	0.03	0.00	0.00	0.00	0.00	0.00	0.05	0.05	0.00	0.03	0.00	0.00	0.00	0.00	0.00	0.00	0.05	0.03	0.03	0.05	0.00	0.00	0.00	0.00
15/09/2011	September	Zooplankton	*Daphnia*	0.09	0.01	0.07	0.01	2.53	0.03	0.17	0.02	0.86	0.00	3.08	0.23	0.11	0.08	0.03	0.02	0.00	0.00	0.00	0.00	0.00	0.04	0.03	0.00	0.02	0.00	0.00	0.00	0.00	0.01	0.00	0.04	0.01	0.02	0.06	0.00	0.00	0.00	0.00
15/09/2011	September	Zooplankton	*Daphnia*	0.45	0.01	0.13	0.00	3.34	0.06	0.20	0.04	0.95	0.00	3.27	0.21	0.07	0.09	0.00	0.01	0.00	0.00	0.00	0.00	0.00	0.02	0.02	0.00	0.01	0.00	0.00	0.00	0.00	0.01	0.00	0.10	0.04	0.04	0.00	0.00	0.00	0.00	0.00
26/10/2011	October	Zooplankton	*Daphnia*	0.03	0.00	0.02	0.00	1.27	0.60	0.10	0.02	1.39	0.00	2.94	0.58	0.54	0.14	0.35	0.03	0.00	0.00	0.02	0.00	0.02	0.58	1.36	0.00	0.02	0.00	0.00	0.03	0.00	0.02	0.00	0.02	0.00	0.00	0.03	0.00	0.00	0.00	0.00
26/10/2011	October	Zooplankton	*Daphnia*	0.00	0.00	0.01	0.00	0.94	0.45	0.07	0.01	0.85	0.00	2.85	0.22	0.12	0.10	0.00	0.02	0.00	0.00	0.00	0.00	0.00	0.06	0.06	0.00	0.02	0.00	0.00	0.00	0.00	0.02	0.00	0.00	0.00	0.00	0.00	0.00	0.00	0.00	0.00
12/12/2011	December	Zooplankton	*Daphnia*	0.00	0.01	0.01	0.00	0.86	0.01	0.07	0.02	0.74	0.00	3.94	0.56	0.41	0.07	0.14	0.01	0.01	0.01	0.01	0.00	0.01	0.25	0.46	0.00	0.02	0.01	0.00	0.01	0.00	0.01	0.01	0.00	0.00	0.01	0.00	0.00	0.01	0.01	0.00
12/12/2011	December	Zooplankton	*Daphnia*	0.05	0.01	0.02	0.01	1.52	1.00	0.10	0.03	0.92	0.00	4.59	0.66	0.43	0.07	0.14	0.01	0.01	0.02	0.01	0.00	0.01	0.17	0.27	0.00	0.02	0.01	0.00	0.00	0.00	0.01	0.01	0.01	0.01	0.00	0.00	0.00	0.01	0.01	0.00
19/01/2012	January	Zooplankton	*Daphnia*	0.19	0.02	0.09	0.00	2.46	0.21	0.12	0.05	1.20	0.00	3.92	0.12	0.04	0.11	0.00	0.02	0.00	0.00	0.00	0.00	0.00	0.02	0.02	0.00	0.02	0.00	0.00	0.00	0.00	0.02	0.00	0.04	0.02	0.00	0.00	0.00	0.00	0.00	0.00
29/02/2012	February	Zooplankton	*Daphnia*	0.38	0.03	0.10	0.00	2.04	0.80	0.13	0.08	1.22	0.00	3.31	0.21	0.08	0.16	0.00	0.03	0.00	0.00	0.00	0.00	0.00	0.03	0.05	0.00	0.03	0.00	0.00	0.00	0.00	0.00	0.00	0.08	0.03	0.00	0.00	0.00	0.00	0.00	0.00
29/02/2012	February	Zooplankton	*Daphnia*	0.60	0.02	0.15	0.00	2.75	1.59	0.17	0.09	1.55	0.00	3.56	0.43	0.26	0.15	0.17	0.02	0.00	0.00	0.00	0.00	0.00	0.13	0.24	0.00	0.02	0.00	0.00	0.02	0.00	0.02	0.02	0.13	0.06	0.00	0.00	0.00	0.02	0.00	0.00
04/04/2012	April	Zooplankton	*Daphnia*	0.79	0.01	0.20	0.00	3.33	2.95	0.00	0.01	1.27	0.01	4.08	0.68	0.53	0.15	0.54	0.03	0.00	0.00	0.01	0.00	0.01	0.20	0.53	0.00	0.03	0.00	0.00	0.03	0.00	0.01	0.01	0.31	0.16	0.00	0.00	0.00	0.01	0.01	0.00
23/05/2012	May	Zooplankton	*Daphnia*	2.05	0.01	0.15	0.02	5.46	4.86	0.00	0.03	1.16	0.01	3.32	2.51	1.29	0.42	1.68	0.03	0.00	0.00	0.01	0.01	0.02	1.72	3.33	0.00	0.02	0.00	0.00	0.08	0.00	0.01	0.00	0.14	0.08	0.00	0.06	0.06	0.01	0.00	0.00
23/05/2012	May	Zooplankton	*Daphnia*	1.14	0.00	0.10	0.00	4.12	3.02	0.00	0.02	1.23	0.00	2.71	1.87	0.89	0.33	1.02	0.03	0.00	0.00	0.02	0.00	0.02	1.19	2.19	0.00	0.02	0.00	0.00	0.05	0.00	0.02	0.00	0.07	0.03	0.00	0.05	0.03	0.00	0.00	0.00
19/05/2011	May	Zooplankton	*Leptodiaptomus minutus*	1.92	0.02	0.14	0.00	4.63	1.13	0.00	0.03	1.21	0.00	2.64	1.27	2.09	0.00	3.70	0.06	0.00	0.00	0.08	0.02	0.02	0.50	3.24	0.00	0.05	0.02	0.00	7.83	0.00	0.02	0.28	0.14	0.08	0.00	0.08	0.09	0.05	0.00	0.00
19/05/2011	May	Zooplankton	*Leptodiaptomus minutus*	1.66	0.02	0.11	0.00	4.23	0.98	0.00	0.02	1.15	0.02	2.29	1.08	1.76	0.00	3.13	0.06	0.00	0.00	0.08	0.00	0.02	0.40	2.65	0.00	0.04	0.02	0.00	6.47	0.00	0.00	0.25	0.11	0.08	0.00	0.06	0.08	0.04	0.00	0.00
22/06/2011	June	Zooplankton	*Leptodiaptomus minutus*	3.64	0.03	0.22	0.00	7.40	1.94	0.00	0.03	3.01	0.00	2.26	2.12	1.81	0.00	2.23	0.14	0.00	0.00	0.08	0.00	0.03	0.64	1.56	0.00	0.14	0.00	0.00	1.68	0.00	0.03	0.06	0.25	0.08	0.00	0.11	0.08	0.03	0.00	0.00
22/06/2011	June	Zooplankton	*Leptodiaptomus minutus*	2.11	0.02	0.17	0.00	5.96	1.83	0.00	0.02	2.18	0.00	2.25	2.39	1.84	0.00	2.13	0.14	0.00	0.05	0.07	0.02	0.02	0.74	1.92	0.00	0.12	0.02	0.00	2.36	0.00	0.02	0.07	0.17	0.07	0.00	0.10	0.10	0.02	0.00	0.00
13/07/2011	July	Zooplankton	*Leptodiaptomus minutus*	0.81	0.00	0.13	0.00	5.16	1.03	0.18	0.02	1.63	0.02	1.43	1.13	1.41	0.24	1.85	0.07	0.00	0.00	0.06	0.02	0.02	0.60	1.26	0.02	0.15	0.02	0.00	1.68	0.02	0.04	0.17	0.09	0.04	0.04	0.00	0.07	0.02	0.00	0.00
13/07/2011	July	Zooplankton	*Leptodiaptomus minutus*	3.98	0.02	0.38	0.00	9.01	2.86	0.32	0.05	2.44	0.02	2.69	2.29	3.33	0.40	4.71	0.14	0.00	0.07	0.12	0.03	0.05	1.33	3.11	0.02	0.19	0.02	0.00	3.81	0.02	0.03	0.00	0.38	0.13	0.08	0.18	0.15	0.03	0.00	0.00
17/08/2011	August	Zooplankton	*Leptodiaptomus minutus*	4.71	0.01	0.64	0.00	13.09	5.04	0.55	0.01	3.84	0.06	5.44	4.40	5.52	0.01	6.37	0.38	0.00	0.16	0.23	0.03	0.12	2.15	4.12	0.06	0.50	0.05	0.02	3.95	0.05	0.06	0.27	0.71	0.22	0.21	0.45	0.25	0.07	0.01	0.00
17/08/2011	August	Zooplankton	*Leptodiaptomus minutus*	4.44	0.00	0.45	0.00	9.12	2.98	0.34	0.03	2.47	0.03	2.99	2.32	2.56	0.43	2.59	0.15	0.00	0.00	0.09	0.03	0.06	1.04	2.05	0.03	0.18	0.03	0.00	2.37	0.03	0.03	0.15	0.48	0.15	0.12	0.24	0.00	0.03	0.00	0.00
15/09/2011	September	Zooplankton	*Leptodiaptomus minutus*	0.43	0.00	0.07	0.00	2.84	0.02	0.15	0.00	1.22	0.00	1.18	0.58	0.64	0.17	0.84	0.06	0.00	0.00	0.04	0.00	0.02	0.47	1.50	0.02	0.08	0.00	0.00	2.89	0.00	0.02	0.13	0.06	0.02	0.02	0.06	0.00	0.02	0.00	0.00
15/09/2011	September	Zooplankton	*Leptodiaptomus minutus*	0.98	0.00	0.11	0.00	3.61	0.72	0.19	0.00	1.27	0.00	1.31	0.68	0.78	0.19	1.08	0.06	0.00	0.00	0.04	0.00	0.02	0.51	1.60	0.02	0.08	0.00	0.00	2.73	0.00	0.02	0.12	0.11	0.04	0.04	0.08	0.00	0.02	0.00	0.00
26/10/2011	October	Zooplankton	*Leptodiaptomus minutus*	3.96	0.04	0.38	0.02	7.73	4.18	0.01	0.06	1.60	0.02	3.14	2.63	4.56	0.49	10.31	0.27	0.00	0.11	0.23	0.02	0.06	1.55	5.86	0.02	0.22	0.05	0.01	6.52	0.02	0.02	0.33	0.45	0.19	0.13	0.22	0.22	0.05	0.00	0.00
26/10/2011	October	Zooplankton	*Leptodiaptomus minutus*	3.50	0.03	0.33	0.00	7.47	3.99	0.01	0.07	1.65	0.02	3.31	2.81	4.97	0.53	11.25	0.29	0.00	0.13	0.27	0.03	0.07	1.80	6.84	0.02	0.24	0.06	0.01	8.09	0.03	0.04	0.39	0.40	0.17	0.13	0.22	0.23	0.06	0.00	0.00

26/10/2011	October	Zooplankton	*Leptodiaptomus minutus*	0.62	0.01	0.17	0.01	6.03	2.88	0.01	0.05	1.73	0.04	3.41	2.68	4.59	0.48	9.75	0.33	0.00	0.15	0.27	0.03	0.08	1.56	5.47	0.03	0.30	0.07	0.01	5.70	0.04	0.04	0.41	0.15	0.07	0.09	0.21	0.22	0.05	0.01	0.00
12/12/2011	December	Zooplankton	*Leptodiaptomus minutus*	2.60	0.02	0.30	0.01	7.99	4.77	0.01	0.02	2.23	0.05	5.76	5.18	6.91	0.78	16.49	0.44	0.01	0.08	0.42	0.05	0.17	2.54	9.20	0.05	0.37	0.08	0.02	10.47	0.06	0.08	0.57	0.37	0.17	0.16	0.27	0.31	0.08	0.01	0.00
12/12/2011	December	Zooplankton	*Leptodiaptomus minutus*	2.75	0.03	0.28	0.01	7.53	4.54	0.01	0.02	1.73	0.03	5.58	4.62	6.90	0.78	16.46	0.41	0.01	0.08	0.42	0.05	0.17	2.56	9.34	0.04	0.38	0.08	0.02	10.55	0.05	0.08	0.59	0.35	0.16	0.15	0.26	0.31	0.08	0.01	0.00
19/01/2012	January	Zooplankton	*Leptodiaptomus minutus*	1.22	0.01	0.24	0.00	8.14	4.19	0.01	0.11	2.06	0.06	6.25	5.41	8.22	0.82	17.98	0.45	0.01	0.08	0.46	0.05	0.17	2.86	10.78	0.05	0.42	0.09	0.02	11.40	0.07	0.10	0.68	0.24	0.10	0.14	0.27	0.37	0.09	0.01	0.00
19/01/2012	January	Zooplankton	*Leptodiaptomus minutus*	9.85	0.07	0.61	0.02	11.42	7.56	0.01	0.03	1.93	0.03	6.89	5.82	8.95	0.95	20.04	0.43	0.01	0.08	0.46	0.05	0.18	2.86	10.79	0.05	0.39	0.09	0.02	11.03	0.06	0.08	0.65	0.97	0.41	0.27	0.36	0.40	0.10	0.01	0.00
29/02/2012	February	Zooplankton	*Leptodiaptomus minutus*	8.69	0.03	0.60	0.02	11.72	7.71	0.01	0.03	2.18	0.08	7.57	6.34	9.36	0.94	18.97	0.46	0.01	0.10	0.50	0.06	0.01	3.09	11.11	0.05	0.45	0.11	0.02	10.82	0.06	0.08	0.77	0.95	0.40	0.28	0.38	0.42	0.11	0.01	0.00
04/04/2012	April	Zooplankton	*Leptodiaptomus minutus*	7.39	0.03	0.48	0.02	9.08	0.26	0.02	0.11	1.72	0.02	5.79	4.38	6.00	0.54	9.22	0.26	0.01	0.18	0.30	0.03	0.11	1.99	7.32	0.03	0.28	0.06	0.02	7.14	0.04	0.05	0.58	0.82	0.33	0.20	0.28	0.28	0.08	0.01	0.00
04/04/2012	April	Zooplankton	*Leptodiaptomus minutus*	5.97	0.03	0.43	0.02	8.55	0.26	0.02	0.09	1.66	0.06	5.42	4.08	5.54	0.49	8.52	0.24	0.01	0.16	0.27	0.03	0.09	1.87	6.91	0.03	0.26	0.05	0.01	6.74	0.03	0.04	0.55	0.69	0.28	0.18	0.25	0.25	0.08	0.01	0.00
23/05/2012	May	Zooplankton	*Leptodiaptomus minutus*	1.95	0.00	0.11	0.00	4.39	1.59	0.00	0.01	1.31	0.01	1.89	1.76	1.19	0.00	1.37	0.06	0.00	0.00	0.10	0.01	0.02	1.13	2.66	0.01	0.07	0.01	0.00	5.30	0.01	0.01	0.22	0.17	0.10	0.00	0.07	0.00	0.04	0.00	0.00
23/05/2012	May	Zooplankton	*Leptodiaptomus minutus*	2.21	0.00	0.11	0.00	4.90	1.81	0.01	0.02	1.70	0.02	2.16	2.07	1.44	0.17	1.70	0.08	0.00	0.03	0.11	0.01	0.03	1.26	2.93	0.01	0.09	0.01	0.00	5.58	0.01	0.01	0.23	0.18	0.10	0.00	0.08	0.00	0.05	0.00	0.00
19/05/2011	May	Zooplankton	*Mesocyclops edax*	1.71	0.00	0.24	0.00	5.18	2.01	0.00	0.02	1.68	0.02	2.09	0.97	1.14	0.00	1.14	0.07	0.05	0.00	0.05	0.10	0.05	0.41	1.85	0.00	0.05	0.02	0.00	3.01	0.02	0.05	0.12	0.17	0.07	0.00	0.15	0.00	0.02	0.00	0.00
19/05/2011	May	Zooplankton	*Mesocyclops edax*	0.95	0.00	0.15	0.00	4.99	0.20	0.25	0.05	2.33	0.05	1.87	0.66	0.71	0.35	0.56	0.05	0.05	0.00	0.05	0.05	0.05	0.25	0.92	0.00	0.05	0.00	0.00	1.17	0.00	0.05	0.05	0.15	0.05	0.05	0.15	0.05	0.00	0.00	0.00
22/06/2011	June	Zooplankton	*Mesocyclops edax*	3.02	0.02	0.29	0.00	6.52	2.68	0.31	0.04	2.51	0.05	3.10	1.85	1.45	0.01	0.99	0.15	0.05	0.11	0.12	0.10	0.05	0.72	1.14	0.02	0.12	0.02	0.01	0.98	0.01	0.03	0.05	0.27	0.10	0.00	0.18	0.00	0.04	0.01	0.00
22/06/2011	June	Zooplankton	*Mesocyclops edax*	2.55	0.01	0.29	0.00	6.25	2.61	0.32	0.05	2.51	0.04	3.28	2.32	1.87	0.01	1.42	0.20	0.06	0.15	0.19	0.18	0.08	1.14	2.07	0.03	0.19	0.04	0.01	2.62	0.02	0.06	0.15	0.27	0.09	0.00	0.17	0.00	0.04	0.01	0.00
13/07/2011	July	Zooplankton	*Mesocyclops edax*	1.16	0.00	0.58	0.03	5.30	0.94	0.56	0.05	1.89	0.03	1.53	1.15	1.81	0.00	0.74	0.08	0.03	0.05	0.05	0.10	0.03	0.20	1.20	0.00	0.05	0.03	0.00	1.15	0.00	0.03	0.03	0.15	0.05	0.00	0.13	0.00	0.00	0.00	0.00
13/07/2011	July	Zooplankton	*Mesocyclops edax*	1.10	0.03	0.32	0.00	5.55	0.22	0.35	0.03	2.07	0.03	1.82	0.82	0.87	0.22	0.33	0.08	0.03	0.05	0.05	0.05	0.03	0.16	0.30	0.00	0.08	0.03	0.00	0.25	0.00	0.03	0.06	0.14	0.05	0.00	0.14	0.00	0.03	0.00	0.00
17/08/2011	August	Zooplankton	*Mesocyclops edax*	0.35	0.00	0.32	0.00	4.62	0.36	0.59	0.04	1.99	0.00	1.27	0.60	1.07	0.00	0.32	0.04	0.00	0.00	0.04	0.08	0.00	0.20	0.80	0.00	0.04	0.00	0.00	0.96	0.00	0.04	0.04	0.08	0.04	0.00	0.08	0.00	0.00	0.00	0.00
17/08/2011	August	Zooplankton	*Mesocyclops edax*	1.01	0.00	0.44	0.00	5.18	0.27	0.58	0.07	2.08	0.00	1.70	0.61	1.16	0.00	0.37	0.07	0.00	0.00	0.03	0.07	0.03	0.31	0.75	0.00	0.03	0.00	0.00	0.79	0.00	0.03	0.03	0.14	0.07	0.07	0.14	0.00	0.00	0.00	0.00
15/09/2011	September	Zooplankton	*Mesocyclops edax*	1.02	0.00	0.24	0.00	5.97	1.11	0.28	0.04	2.70	0.00	1.55	0.44	0.24	0.28	0.12	0.08	0.00	0.00	0.04	0.00	0.00	0.08	0.08	0.00	0.08	0.04	0.00	0.08	0.00	0.04	0.04	0.12	0.04	0.04	0.12	0.00	0.00	0.00	0.00
15/09/2011	September	Zooplankton	*Mesocyclops edax*	0.21	0.01	0.11	0.00	3.64	1.51	0.37	0.04	2.00	0.03	2.83	0.82	0.56	0.09	0.26	0.15	0.05	0.06	0.05	0.03	0.02	0.38	0.34	0.02	0.14	0.02	0.00	0.32	0.02	0.06	0.07	0.06	0.02	0.06	0.19	0.00	0.02	0.02	0.00
23/05/2012	May	Zooplankton	*Mesocyclops edax*	2.96	0.01	0.23	0.00	5.92	2.55	0.01	0.03	1.93	0.04	2.50	2.12	1.73	0.00	2.41	0.13	0.02	0.09	0.17	0.13	0.07	1.46	3.64	0.02	0.13	0.03	0.01	6.12	0.03	0.06	0.19	0.20	0.11	0.00	0.13	0.08	0.05	0.01	0.00
23/05/2012	May	Zooplankton	*Mesocyclops edax*	2.40	0.01	0.19	0.00	5.33	2.12	0.01	0.03	1.78	0.03	2.09	1.78	1.46	0.00	1.97	0.09	0.01	0.07	0.13	0.10	0.05	1.12	2.97	0.01	0.10	0.03	0.01	4.95	0.02	0.05	0.17	0.16	0.09	0.00	0.10	0.07	0.04	0.01	0.00
06/07/2011	July	Seston		0.41	0.01	0.09	0.00	1.43	1.44	0.04	0.01	0.48	0.00	0.22	0.20	0.43	0.03	0.18	0.03	0.01	0.00	0.01	0.01	0.00	0.09	0.43	0.00	0.04	0.00	0.00	0.09	0.01	0.07	0.01	0.15	0.07	0.01	0.03	0.00	0.00	0.00	0.06
06/07/2011	July	Seston		0.63	0.02	0.14	0.00	2.82	2.22	0.07	0.01	0.78	0.00	0.69	0.56	0.72	0.08	0.55	0.06	0.03	0.00	0.02	0.01	0.01	0.16	0.49	0.01	0.09	0.01	0.00	0.25	0.02	0.11	0.08	0.21	0.12	0.01	0.05	0.00	0.00	0.00	0.19
06/07/2011	July	Seston		0.58	0.02	0.11	0.00	2.32	1.53	0.05	0.01	0.63	0.00	0.39	0.34	0.48	0.05	0.30	0.03	0.02	0.00	0.01	0.00	0.00	0.07	0.21	0.00	0.05	0.01	0.00	0.10	0.01	0.07	0.01	0.15	0.08	0.01	0.03	0.00	0.00	0.00	0.10
06/07/2011	July	Seston		0.51	0.02	0.10	0.00	1.47	1.45	0.04	0.01	0.41	0.00	0.21	0.15	0.32	0.03	0.15	0.02	0.01	0.00	0.01	0.00	0.00	0.06	0.31	0.00	0.03	0.00	0.00	0.06	0.01	0.06	0.00	0.17	0.08	0.01	0.02	0.00	0.00	0.00	0.05
08/09/2011	September	Seston		0.16	0.01	0.06	0.00	0.77	0.88	0.02	0.00	0.28	0.00	0.07	0.06	0.14	0.02	0.04	0.02	0.00	0.00	0.00	0.00	0.00	0.04	0.07	0.00	0.03	0.00	0.00	0.04	0.01	0.04	0.00	0.10	0.05	0.00	0.02	0.00	0.00	0.00	0.02
08/09/2011	September	Seston		0.38	0.02	0.09	0.00	1.17	1.55	0.04	0.01	0.51	0.00	0.14	0.10	0.21	0.03	0.08	0.02	0.01	0.00	0.01	0.00	0.00	0.06	0.11	0.01	0.04	0.00	0.00	0.06	0.01	0.06	0.02	0.19	0.10	0.01	0.03	0.00	0.00	0.00	0.03
08/09/2011	September	Seston		0.69	0.02	0.10	0.00	1.93	1.35	0.04	0.01	0.62	0.00	0.37	0.22	0.65	0.05	0.62	0.03	0.01	0.00	0.01	0.01	0.00	0.09	0.23	0.01	0.06	0.00	0.00	0.13	0.01	0.08	0.05	0.23	0.13	0.01	0.04	0.00	0.00	0.00	0.21
08/09/2011	September	Seston		0.56	0.02	0.07	0.00	1.65	1.19	0.04	0.01	0.56	0.00	0.40	0.21	0.62	0.05	0.61	0.03	0.01	0.00	0.01	0.01	0.00	0.09	0.23	0.01	0.06	0.00	0.00	0.15	0.01	0.07	0.01	0.20	0.11	0.01	0.04	0.00	0.00	0.00	0.21
04/12/2011	December	Seston		0.55	0.02	0.17	0.00	1.73	1.22	0.06	0.01	0.47	0.00	0.50	0.26	0.39	0.04	0.38	0.05	0.01	0.00	0.01	0.01	0.01	0.05	0.25	0.01	0.09	0.01	0.01	0.14	0.02	0.10	0.04	0.18	0.13	0.01	0.04	0.00	0.00	0.00	0.14
04/12/2011	December	Seston		5.84	0.09	0.74	0.04	8.52	6.92	0.01	0.06	1.10	0.00	6.07	1.50	1.81	0.14	2.07	0.19	0.00	0.07	0.03	0.01	0.01	0.19	0.95	0.01	0.25	0.23	0.00	0.43	0.01	0.07	0.02	0.05	0.08	0.06	0.07	0.00	0.01	0.01	0.00
04/12/2011	December	Seston		5.37	0.09	0.73	0.04	8.10	6.36	0.01	0.05	1.11	0.00	5.63	1.48	1.82	0.14	2.04	0.19	0.00	0.07	0.03	0.01	0.01	0.20	1.00	0.01	0.26	0.18	0.00	0.48	0.01	0.08	0.02	0.09	0.08	0.06	0.07	0.00	0.01	0.01	0.00
22/02/2012	February	Seston		0.03	0.00	0.03	0.00	0.98	0.70	0.04	0.02	0.67	0.01	0.41	0.14	0.20	0.06	0.21	0.06	0.01	0.00	0.01	0.01	0.00	0.07	0.27	0.01	0.16	0.01	0.01	0.27	0.03	0.14	0.10	0.05	0.03	0.00	0.04	0.01	0.00	0.00	0.07
22/02/2012	February	Seston		0.09	0.01	0.05	0.00	1.07	0.83	0.04	0.02	0.67	0.01	0.38	0.11	0.20	0.05	0.22	0.05	0.01	0.00	0.01	0.01	0.00	0.05	0.22	0.01	0.14	0.01	0.00	0.19	0.02	0.14	0.08	0.08	0.06	0.00	0.04	0.01	0.00	0.00	0.08
22/02/2012	February	Seston		0.07	0.00	0.06	0.00	1.11	0.66	0.04	0.02	0.76	0.00	0.10	0.09	0.06	0.03	0.05	0.05	0.00	0.00	0.00	0.00	0.00	0.03	0.09	0.01	0.09	0.00	0.00	0.07	0.01	0.09	0.01	0.07	0.00	0.00	0.03	0.00	0.00	0.00	0.02
22/02/2012	February	Seston		0.19	0.01	0.12	0.00	1.73	1.47	0.07	0.04	0.90	0.01	0.44	0.17	0.11	0.05	0.11	0.07	0.01	0.00	0.01	0.00	0.00	0.06	0.20	0.02	0.12	0.01	0.00	0.17	0.03	0.14	0.05	0.19	0.13	0.00	0.06	0.01	0.00	0.00	0.04
22/02/2012	February	Seston		0.23	0.02	0.07	0.01	1.12	0.52	0.02	0.00	0.54	0.09	0.13	0.06	0.02	0.03	0.00	0.01	0.00	0.00	0.00	0.00	0.00	0.00	0.02	0.00	0.01	0.00	0.00	0.00	0.00	0.01	0.00	0.02	0.02	0.00	0.00	0.00	0.00	0.00	0.00
22/02/2012	February	Seston		1.76	0.05	0.54	0.02	6.19	0.65	0.00	0.03	1.89	0.00	2.71	0.38	0.23	0.07	0.14	0.11	0.00	0.00	0.00	0.00	0.00	0.03	0.12	0.00	0.22	0.11	0.00	0.08	0.04	0.13	0.01	0.01	0.03	0.01	0.04	0.00	0.00	0.00	0.00
22/02/2012	February	Seston		1.88	0.03	0.42	0.02	6.05	0.73	0.00	0.03	1.60	0.00	2.51	0.31	0.24	0.08	0.18	0.11	0.00	0.00	0.00	0.00	0.00	0.03	0.14	0.00	0.22	0.10	0.00	0.09	0.05	0.11	0.00	0.01	0.04	0.00	0.03	0.00	0.01	0.00	0.00
22/02/2012	February	Seston		3.52	0.10	0.76	0.03	6.69	3.69	0.01	0.04	1.29	0.00	2.84	0.34	0.11	0.06	0.00	0.07	0.00	0.00	0.00	0.00	0.00	0.02	0.07	0.00	0.11	0.00	0.00	0.04	0.01	0.04	0.00	0.02	0.02	0.01	0.05	0.00	0.00	0.01	0.00
22/02/2012	February	Seston		9.58	0.31	2.49	0.09	21.87	12.76	0.00	0.13	6.61	0.00	7.05	0.76	0.25	0.19	0.00	0.29	0.00	0.00	0.00	0.00	0.00	0.06	0.16	0.03	0.42	0.00	0.00	0.06	0.03	0.19	0.00	0.06	0.09	0.03	0.13	0.00	0.00	0.00	0.00
03/04/2012	April	Seston		0.94	0.02	0.14	0.00	1.87	0.05	0.00	0.04	0.43	0.00	0.87	0.18	0.22	0.04	0.32	0.06	0.00	0.00	0.00	0.00	0.00	0.02	0.12	0.00	0.06	0.00	0.00	0.07	0.01	0.04	0.00	0.00	0.02	0.00	0.03	0.00	0.00	0.00	0.00
03/04/2012	April	Seston		0.78	0.02	0.12	0.01	1.59	0.05	0.00	0.03	0.38	0.00	0.75	0.14	0.19	0.04	0.29	0.05	0.00	0.00	0.00	0.00	0.00	0.03	0.11	0.00	0.06	0.01	0.00	0.07	0.01	0.04	0.00	0.01	0.10	0.01	0.03	0.01	0.00	0.00	0.00
03/04/2012	April	Seston		0.89	0.02	0.16	0.00	1.75	0.22	0.00	0.03	0.45	0.00	0.69	0.10	0.12	0.04	0.09	0.03	0.00	0.00	0.00	0.00	0.00	0.01	0.06	0.00	0.04	0.00	0.00	0.03	0.01	0.03	0.00	0.01	0.02	0.01	0.03	0.00	0.00	0.00	0.00
03/04/2012	April	Seston		0.92	0.03	0.14	0.01	1.95	0.13	0.05	0.01	0.38	0.01	0.46	0.06	0.05	0.05	0.00	0.03	0.00	0.00	0.00	0.00	0.00	0.01	0.03	0.00	0.05	0.00	0.00	0.01	0.01	0.03	0.00	0.01	0.03	0.01	0.00	0.00	0.01	0.00	0.00
20/04/2012	April	Seston		0.65	0.01	0.06	0.01	1.25	0.79	0.02	0.00	0.33	0.07	0.26	0.10	0.33	0.06	0.42	0.03	0.00	0.00	0.00	0.00	0.00	0.03	0.13	0.00	0.01	0.00	0.00	0.13	0.01	0.01	0.00	0.05	0.03	0.01	0.01	0.00	0.00	0.00	0.00
20/04/2012	April	Seston		7.05	0.09	0.68	0.03	12.74	7.36	0.01	0.08	1.96	0.00	6.13	1.60	3.25	0.01	3.15	0.35	0.00	0.08	0.01	0.00	0.01	0.12	0.66	0.01	0.38	0.06	0.00	0.67	0.03	0.19	0.03	0.01	0.05	0.01	0.07	0.00	0.01	0.00	0.00
20/04/2012	April	Seston		6.96	0.08	0.60	0.01	12.47	7.53	0.01	0.08	1.59	0.00	6.20	1.63	3.47	0.01	3.43	0.35	0.00	0.08	0.01	0.01	0.01	0.12	0.73	0.01	0.37	0.06	0.00	0.73	0.09	0.20	0.03	0.01	0.06	0.01	0.06	0.00	0.00	0.01	0.00
20/04/2012	April	Seston		6.30	0.09	0.78	0.03	12.02	6.19	0.01	0.06	1.68	0.00	5.18	1.19	2.41	0.01	2.11	0.28	0.00	0.06	0.01	0.01	0.01	0.08	0.44	0.01	0.32	0.05	0.00	0.41	0.03	0.19	0.00	0.01	0.06	0.01	0.06	0.00	0.01	0.01	0.00
20/04/2012	April	Seston		5.19	0.07	0.70	0.03	10.62	5.07	0.00	0.06	1.87	0.00	4.34	1.00	1.99	0.00	1.66	0.24	0.00	0.05	0.00	0.00	0.00	0.06	0.36	0.01	0.27	0.04	0.00	0.32	0.07	0.17	0.00	0.01	0.05	0.01	0.06	0.00	0.00	0.00	0.00
02/05/2012	May	Seston		0.62	0.04	0.10	0.02	1.19	0.72	0.02	0.00	0.37	0.03	0.32	0.33	0.27	0.05	0.41	0.03	0.00	0.00	0.00	0.00	0.00	0.03	0.17	0.00	0.01	0.00	0.00	0.13	0.00	0.01	0.00	0.04	0.04	0.00	0.00	0.00	0.00	0.00	0.00
02/05/2012	May	Seston		5.42	0.11	0.61	0.03	7.59	6.10	0.01	0.04	1.07	0.00	3.97	1.22	1.74	0.00	2.67	0.27	0.00	0.04	0.01	0.01	0.01	0.19	1.00	0.01	0.21	0.02	0.00	1.00	0.02	0.11	0.00	0.01	0.04	0.01	0.06	0.00	0.01	0.01	0.00
02/05/2012	May	Seston		8.65	0.18	1.05	0.04	13.18	8.08	0.01	0.02	1.26	0.01	5.03	0.95	1.10	0.01	1.46	0.21	0.00	0.00	0.01	0.00	0.00	0.11	0.40	0.01	0.21	0.02	0.00	0.31	0.02	0.13	0.00	0.03	0.04	0.01	0.00	0.00	0.01	0.00	0.00
09/05/2012	May	Seston		0.76	0.10	0.53	0.08	3.98	0.63	0.10	0.00	1.58	0.24	0.35	0.18	0.12	0.08	0.41	0.06	0.00	0.00	0.00	0.00	0.02	0.02	0.12	0.00	0.02	0.00	0.06	0.10	0.00	0.02	0.00	0.08	0.10	0.02	0.02	0.00	0.00	0.00	0.00
09/05/2012	May	Seston		0.43	0.06	0.44	0.06	3.63	0.26	0.12	0.00	1.34	0.00	0.40	0.21	0.02	0.07	0.00	0.05	0.00	0.00	0.00	0.00	0.00	0.01	0.04	0.00	0.02	0.01	0.05	0.02	0.01	0.05	0.00	0.05	0.05	0.01	0.01	0.00	0.00	0.00	0.00
16/05/2012	May	Seston		0.31	0.00	0.06	0.00	1.24	1.72	0.03	0.00	0.31	0.00	0.23	0.30	0.44	0.05	0.61	0.03	0.01	0.00	0.02	0.00	0.00	0.19	0.66	0.01	0.06	0.01	0.00	0.45	0.01	0.06	0.01	0.08	0.00	0.00	0.03	0.00	0.00	0.00	0.21
16/05/2012	May	Seston		0.69	0.01	0.08	0.00	1.64	1.90	0.03	0.01	0.44	0.00	0.30	0.48	0.45	0.06	0.78	0.04	0.01	0.00	0.01	0.00	0.01	0.25	0.66	0.01	0.07	0.00	0.00	0.47	0.01	0.07	0.01	0.13	0.10	0.00	0.04	0.00	0.00	0.00	0.27
16/05/2012	May	Seston		0.55	0.01	0.07	0.00	1.39	2.00	0.03	0.01	0.42	0.00	0.32	0.40	0.48	0.05	0.67	0.04	0.01	0.00	0.02	0.00	0.00	0.19	0.68	0.01	0.06	0.00	0.00	0.45	0.01	0.06	0.01	0.11	0.08	0.00	0.03	0.00	0.00	0.00	0.23

16/05/2012	May	Seston	1.13	0.01	0.10	0.00	1.85	2.29	0.03	0.01	0.46	0.00	0.33	0.51	0.48	0.07	0.81	0.04	0.01	0.00	0.01	0.00	0.01	0.25	0.66	0.01	0.07	0.01	0.00	0.45	0.01	0.07	0.01	0.18	0.13	0.01	0.00	0.00	0.00	0.00	0.28
13/06/2012	June	Seston	4.16	0.04	0.56	0.02	8.50	5.93	0.01	0.04	1.33	0.00	3.00	0.63	0.92	0.00	0.69	0.15	0.00	0.00	0.01	0.00	0.00	0.18	0.45	0.01	0.35	0.02	0.00	0.17	0.03	0.15	0.00	0.06	0.03	0.01	0.05	0.00	0.00	0.00	0.00
13/06/2012	June	Seston	3.79	0.04	0.50	0.02	7.67	5.35	0.00	0.03	1.15	0.00	2.61	0.55	0.85	0.00	0.62	0.12	0.00	0.00	0.01	0.00	0.00	0.16	0.40	0.01	0.30	0.02	0.00	0.14	0.02	0.13	0.00	0.05	0.03	0.01	0.05	0.00	0.00	0.00	0.00
13/06/2012	June	Seston	1.82	0.07	0.30	0.02	3.91	0.36	0.00	0.02	0.64	0.00	1.27	0.23	0.45	0.00	0.30	0.07	0.00	0.00	0.00	0.00	0.00	0.06	0.26	0.00	0.15	0.01	0.00	0.06	0.01	0.07	0.00	0.03	0.05	0.00	0.03	0.00	0.00	0.00	0.00
13/06/2012	June	Seston	1.94	0.03	0.32	0.02	4.30	0.38	0.00	0.02	0.68	0.00	1.36	0.25	0.47	0.00	0.30	0.07	0.00	0.00	0.00	0.00	0.00	0.06	0.26	0.00	0.15	0.01	0.00	0.06	0.00	0.07	0.00	0.03	0.05	0.00	0.03	0.00	0.00	0.00	0.00
04/07/2012	July	Seston	0.07	0.00	0.02	0.01	0.93	0.51	0.02	0.01	0.37	0.02	0.19	0.10	0.21	0.03	0.06	0.01	0.00	0.00	0.00	0.00	0.00	0.02	0.05	0.00	0.01	0.00	0.01	0.00	0.00	0.01	0.00	0.01	0.01	0.00	0.01	0.00	0.00	0.00	0.00
04/07/2012	July	Seston	0.15	0.01	0.03	0.01	1.26	0.53	0.02	0.01	0.36	0.00	0.23	0.14	0.21	0.03	0.09	0.01	0.00	0.00	0.00	0.00	0.00	0.03	0.04	0.00	0.01	0.00	0.00	0.00	0.00	0.01	0.00	0.02	0.01	0.00	0.00	0.01	0.00	0.00	0.00
04/07/2012	July	Seston	2.70	0.03	0.31	0.01	7.67	5.09	0.09	0.03	0.76	0.00	2.36	0.72	0.90	0.00	0.38	0.06	0.00	0.00	0.01	0.01	0.00	0.12	0.21	0.01	0.10	0.00	0.00	0.06	0.01	0.05	0.00	0.03	0.01	0.00	0.03	0.00	0.01	0.00	0.00
04/07/2012	July	Seston	3.92	0.07	0.46	0.02	10.74	6.89	0.02	0.02	0.99	0.00	3.26	0.96	1.11	0.00	0.48	0.08	0.00	0.00	0.01	0.00	0.01	0.16	0.28	0.01	0.14	0.01	0.00	0.08	0.01	0.07	0.00	0.02	0.02	0.01	0.04	0.00	0.01	0.00	0.00
04/07/2012	July	Seston	2.93	0.08	0.39	0.02	7.44	5.59	0.10	0.03	0.72	0.00	1.90	0.46	0.94	0.08	0.24	0.05	0.00	0.00	0.01	0.01	0.00	0.08	0.18	0.01	0.11	0.00	0.00	0.05	0.01	0.05	0.00	0.04	0.03	0.01	0.04	0.00	0.00	0.00	0.00
04/07/2012	July	Seston	2.31	0.09	0.31	0.01	5.38	0.14	0.01	0.03	0.58	0.00	1.37	0.33	0.71	0.00	0.18	0.04	0.00	0.00	0.01	0.01	0.00	0.06	0.14	0.01	0.08	0.00	0.00	0.04	0.01	0.04	0.00	0.04	0.02	0.00	0.03	0.00	0.01	0.00	0.00
20/07/2012	July	Seston	3.29	0.03	0.34	0.02	8.06	4.88	0.01	0.03	0.87	0.00	2.10	0.53	0.85	0.00	0.44	0.08	0.00	0.00	0.01	0.01	0.00	0.09	0.20	0.01	0.09	0.00	0.00	0.07	0.01	0.06	0.00	0.03	0.02	0.00	0.06	0.00	0.01	0.03	0.00
20/07/2012	July	Seston	2.48	0.02	0.26	0.02	6.06	3.74	0.01	0.02	0.68	0.00	1.64	0.41	0.67	0.00	0.35	0.06	0.00	0.00	0.01	0.01	0.00	0.07	0.16	0.01	0.07	0.00	0.00	0.06	0.01	0.05	0.00	0.02	0.01	0.00	0.04	0.00	0.01	0.02	0.00
20/07/2012	July	Seston	1.72	0.03	0.30	0.00	4.30	0.59	0.01	0.07	0.49	0.00	1.11	0.27	0.79	0.00	0.24	0.04	0.00	0.00	0.01	0.01	0.00	0.05	0.15	0.01	0.07	0.00	0.00	0.04	0.01	0.03	0.00	0.02	0.02	0.04	0.04	0.00	0.01	0.01	0.00
20/07/2012	July	Seston	1.81	0.03	0.32	0.01	4.50	0.61	0.02	0.06	0.47	0.00	1.12	0.28	0.80	0.00	0.25	0.04	0.00	0.00	0.01	0.01	0.00	0.05	0.16	0.01	0.07	0.00	0.00	0.04	0.01	0.03	0.00	0.03	0.03	0.05	0.04	0.00	0.01	0.01	0.00
06/08/2012	August	Seston	2.30	0.02	0.25	0.01	8.10	0.63	0.09	0.03	13.73	0.00	1.49	0.38	0.59	0.00	0.27	0.08	0.00	0.00	0.00	0.00	0.00	0.06	0.13	0.01	0.07	0.01	0.00	0.04	0.01	0.04	0.00	0.02	0.01	0.00	0.02	0.00	0.00	0.00	0.00
06/08/2012	August	Seston	2.38	0.01	0.25	0.01	8.67	4.34	0.09	0.03	16.29	0.00	1.55	0.38	0.63	0.00	0.28	0.08	0.00	0.00	0.01	0.01	0.00	0.07	0.13	0.00	0.07	0.00	0.00	0.05	0.01	0.04	0.00	0.02	0.01	0.00	0.02	0.00	0.00	0.00	0.00
06/08/2012	August	Seston	1.86	0.02	0.38	0.01	6.58	0.92	0.01	0.04	7.98	0.00	1.07	0.25	0.67	0.00	0.21	0.05	0.00	0.00	0.01	0.01	0.00	0.05	0.15	0.00	0.06	0.00	0.00	0.04	0.00	0.03	0.00	0.02	0.02	0.00	0.02	0.00	0.00	0.00	0.00
06/08/2012	August	Seston	2.39	0.11	0.59	0.01	7.27	1.02	0.01	0.05	10.08	0.00	1.29	0.29	0.71	0.00	0.23	0.06	0.00	0.00	0.01	0.01	0.00	0.05	0.16	0.01	0.06	0.00	0.00	0.05	0.01	0.03	0.00	0.02	0.02	0.01	0.03	0.00	0.00	0.00	0.00
21/08/2012	August	Seston	1.98	0.04	0.30	0.01	6.80	3.69	0.01	0.02	8.81	0.00	1.78	0.40	0.78	0.00	0.26	0.07	0.00	0.00	0.01	0.01	0.00	0.06	0.15	0.00	0.06	0.01	0.00	0.04	0.01	0.04	0.00	0.01	0.01	0.01	0.02	0.00	0.00	0.00	0.00
21/08/2012	August	Seston	1.91	0.04	0.26	0.01	5.95	3.63	0.01	0.02	4.64	0.00	1.67	0.39	0.74	0.00	0.25	0.06	0.00	0.00	0.01	0.01	0.00	0.05	0.14	0.01	0.06	0.01	0.00	0.05	0.00	0.03	0.00	0.01	0.01	0.00	0.03	0.00	0.01	0.00	0.00
21/08/2012	August	Seston	2.17	0.06	0.59	0.01	5.35	1.90	0.00	0.05	6.52	0.00	1.14	0.29	0.80	0.00	0.29	0.05	0.00	0.00	0.01	0.01	0.00	0.05	0.17	0.00	0.05	0.01	0.00	0.04	0.01	0.03	0.00	0.03	0.03	0.05	0.03	0.00	0.00	0.00	0.00
21/08/2012	August	Seston	2.08	0.04	0.59	0.01	5.18	1.86	0.00	0.05	5.99	0.00	1.09	0.31	0.92	0.00	0.35	0.04	0.00	0.00	0.01	0.00	0.00	0.06	0.19	0.00	0.04	0.01	0.00	0.05	0.00	0.02	0.00	0.03	0.02	0.04	0.03	0.00	0.00	0.00	0.00
05/09/2012	September	Seston	2.35	0.03	0.26	0.01	6.61	0.69	0.02	0.02	4.63	0.00	1.33	0.23	0.50	0.07	0.19	0.06	0.00	0.00	0.00	0.00	0.00	0.05	0.10	0.00	0.06	0.00	0.00	0.04	0.01	0.02	0.00	0.02	0.02	0.03	0.02	0.00	0.01	0.00	0.00
05/09/2012	September	Seston	1.81	0.04	0.20	0.01	4.97	3.54	0.01	0.01	5.18	0.00	1.15	0.21	0.49	0.00	0.18	0.04	0.00	0.00	0.00	0.00	0.00	0.04	0.10	0.00	0.04	0.00	0.00	0.04	0.01	0.02	0.00	0.02	0.01	0.02	0.02	0.00	0.00	0.00	0.00
05/09/2012	September	Seston	1.58	0.02	0.26	0.01	4.35	0.29	0.06	0.02	3.92	0.00	0.84	0.13	0.36	0.07	0.09	0.03	0.00	0.00	0.00	0.00	0.00	0.04	0.10	0.00	0.04	0.00	0.00	0.03	0.01	0.03	0.00	0.02	0.02	0.03	0.02	0.00	0.01	0.00	0.00
05/09/2012	September	Seston	1.34	0.05	0.22	0.01	3.31	0.26	0.01	0.02	1.83	0.00	0.66	0.10	0.28	0.05	0.06	0.03	0.00	0.00	0.00	0.00	0.00	0.03	0.07	0.00	0.03	0.00	0.00	0.02	0.00	0.02	0.00	0.02	0.01	0.00	0.02	0.00	0.01	0.00	0.00
19/09/2012	September	Seston	1.39	0.01	0.13	0.00	3.70	2.92	0.01	0.02	1.84	0.00	1.17	0.24	0.41	0.00	0.15	0.03	0.00	0.00	0.00	0.00	0.00	0.03	0.08	0.00	0.04	0.00	0.00	0.03	0.01	0.02	0.00	0.02	0.01	0.03	0.02	0.00	0.01	0.00	0.00
19/09/2012	September	Seston	1.19	0.01	0.11	0.00	3.17	2.72	0.01	0.02	1.78	0.00	1.04	0.21	0.40	0.00	0.14	0.03	0.00	0.00	0.00	0.00	0.00	0.03	0.08	0.00	0.03	0.00	0.00	0.03	0.00	0.02	0.00	0.01	0.01	0.02	0.01	0.00	0.00	0.00	0.00
03/10/2012	October	Seston	1.58	0.01	0.12	0.00	3.61	0.43	0.01	0.02	1.07	0.00	1.13	0.24	0.48	0.00	0.21	0.03	0.00	0.00	0.00	0.00	0.00	0.03	0.11	0.00	0.04	0.00	0.00	0.04	0.01	0.02	0.00	0.01	0.01	0.00	0.01	0.00	0.01	0.00	0.00
03/10/2012	October	Seston	1.52	0.02	0.13	0.00	3.34	0.39	0.01	0.02	1.26	0.00	1.12	0.25	0.54	0.00	0.25	0.03	0.00	0.00	0.00	0.00	0.00	0.03	0.13	0.00	0.04	0.00	0.00	0.05	0.01	0.02	0.00	0.01	0.01	0.00	0.01	0.00	0.01	0.00	0.00
17/10/2012	October	Seston	1.15	0.02	0.28	0.01	2.47	1.72	0.00	0.01	0.67	0.00	1.07	0.25	0.34	0.00	0.27	0.05	0.00	0.00	0.00	0.00	0.00	0.02	0.14	0.00	0.05	0.00	0.00	0.06	0.00	0.04	0.00	0.00	0.01	0.01	0.01	0.00	0.01	0.00	0.00
17/10/2012	October	Seston	1.87	0.04	0.45	0.02	3.73	2.74	0.00	0.02	1.03	0.00	1.62	0.40	0.53	0.05	0.44	0.07	0.00	0.00	0.00	0.00	0.00	0.04	0.28	0.00	0.07	0.01	0.00	0.11	0.00	0.06	0.00	0.01	0.02	0.01	0.02	0.00	0.01	0.00	0.00
31/10/2012	October	Seston	3.81	0.08	0.56	0.04	4.76	4.51	0.01	0.02	1.21	0.00	2.26	0.43	0.59	0.11	0.82	0.07	0.00	0.00	0.01	0.01	0.01	0.07	0.48	0.00	0.06	0.01	0.00	0.29	0.00	0.03	0.01	0.02	0.03	0.01	0.03	0.00	0.00	0.00	0.00
31/10/2012	October	Seston	3.75	0.06	0.37	0.02	4.39	1.44	0.01	0.01	1.31	0.00	1.98	0.42	0.64	0.12	0.89	0.07	0.00	0.00	0.01	0.01	0.01	0.07	0.50	0.00	0.06	0.01	0.00	0.27	0.00	0.02	0.00	0.01	0.03	0.01	0.02	0.00	0.00	0.00	0.00
14/11/2012	November	Seston	5.03	0.05	0.43	0.03	6.55	11.10	0.01	0.02	1.26	0.00	3.13	0.80	0.98	0.13	1.59	0.09	0.00	0.00	0.01	0.01	0.01	0.14	0.99	0.01	0.08	0.01	0.00	0.43	0.01	0.04	0.00	0.01	0.04	0.01	0.03	0.00	0.01	0.00	0.00
14/11/2012	November	Seston	4.53	0.04	0.25	0.00	5.88	0.53	0.01	0.02	1.08	0.00	2.81	0.74	1.02	0.13	1.67	0.09	0.00	0.00	0.01	0.01	0.01	0.14	1.08	0.01	0.08	0.01	0.00	0.53	0.01	0.04	0.00	0.01	0.04	0.03	0.03	0.00	0.00	0.00	0.00
09/01/2013	January	Seston	3.22	0.05	0.35	0.03	3.41	0.38	0.00	0.02	1.44	0.00	1.46	0.35	1.11	0.00	2.02	0.06	0.00	0.00	0.01	0.00	0.00	0.08	0.40	0.01	0.08	0.00	0.01	0.29	0.01	0.05	0.00	0.01	0.04	0.01	0.03	0.00	0.00	0.00	0.00
09/01/2013	January	Seston	2.74	0.05	0.36	0.03	2.95	0.35	0.00	0.02	0.96	0.00	1.28	0.31	0.98	0.00	1.77	0.05	0.00	0.00	0.01	0.00	0.00	0.08	0.38	0.01	0.07	0.00	0.00	0.28	0.01	0.05	0.00	0.01	0.03	0.01	0.02	0.00	0.00	0.00	0.00
09/01/2013	January	Seston	1.76	0.03	0.28	0.02	3.35	0.26	0.00	0.02	0.99	0.00	1.09	0.22	0.34	0.05	0.41	0.06	0.00	0.00	0.01	0.00	0.00	0.05	0.34	0.01	0.08	0.01	0.01	0.12	0.01	0.05	0.00	0.01	0.03	0.01	0.02	0.00	0.00	0.00	0.00
09/01/2013	January	Seston	1.91	0.04	0.30	0.02	3.42	0.29	0.00	0.02	1.01	0.00	1.10	0.21	0.34	0.06	0.41	0.06	0.00	0.00	0.01	0.00	0.00	0.05	0.34	0.01	0.08	0.01	0.00	0.11	0.01	0.05	0.00	0.01	0.03	0.01	0.02	0.00	0.00	0.00	0.00
06/02/2013	February	Seston	2.43	0.04	0.25	0.02	3.43	0.19	0.00	0.02	1.36	0.00	1.45	0.40	0.68	0.00	0.92	0.05	0.00	0.00	0.00	0.00	0.00	0.04	0.22	0.00	0.04	0.00	0.00	0.15	0.01	0.02	0.00	0.01	0.02	0.01	0.02	0.00	0.00	0.00	0.00
06/02/2013	February	Seston	2.49	0.05	0.32	0.03	3.74	0.23	0.00	0.02	1.09	0.00	1.63	0.43	0.69	0.00	0.92	0.05	0.00	0.00	0.00	0.00	0.00	0.04	0.24	0.00	0.04	0.01	0.00	0.16	0.00	0.02	0.00	0.01	0.02	0.01	0.02	0.00	0.00	0.00	0.00
06/02/2013	February	Seston	1.53	0.04	0.31	0.02	3.05	0.44	0.00	0.02	0.77	0.00	0.97	0.24	0.25	0.05	0.26	0.05	0.00	0.00	0.00	0.00	0.00	0.03	0.09	0.01	0.05	0.01	0.00	0.05	0.01	0.03	0.00	0.01	0.01	0.01	0.02	0.00	0.00	0.00	0.00
06/02/2013	February	Seston	1.44	0.03	0.35	0.02	3.35	0.53	0.00	0.02	1.27	0.00	1.11	0.21	0.25	0.06	0.29	0.05	0.00	0.00	0.00	0.00	0.00	0.02	0.11	0.00	0.06	0.00	0.00	0.06	0.01	0.03	0.00	0.01	0.02	0.01	0.02	0.00	0.00	0.00	0.00
06/03/2013	March	Seston	5.21	0.05	0.47	0.04	6.27	0.86	0.00	0.05	1.62	0.00	2.28	1.50	2.07	0.00	3.78	0.11	0.00	0.00	0.05	0.01	0.01	0.36	1.77	0.01	0.10	0.00	0.00	1.25	0.01	0.00	0.04	0.01	0.20	0.01	0.12	0.00	0.02	0.00	0.00
06/03/2013	March	Seston	2.98	0.04	0.36	0.03	4.19	0.81	0.00	0.03	1.23	0.00	1.41	0.50	0.81	0.10	1.44	0.06	0.00	0.00	0.01	0.00	0.00	0.12	0.55	0.00	0.05	0.00	0.00	0.33	0.00	0.02	0.00	0.01	0.03	0.01	0.05	0.00	0.01	0.00	0.00
06/03/2013	March	Seston	3.92	0.03	0.32	0.02	3.71	1.06	0.00	0.03	1.49	0.00	1.27	0.25	1.15	0.13	3.07	0.05	0.00	0.00	0.00	0.00	0.00	0.07	0.45	0.00	0.05	0.00	0.00	0.34	0.00	0.02	0.00	0.02	0.02	0.00	0.02	0.00	0.00	0.00	0.00
06/03/2013	March	Seston	3.03	0.04	0.25	0.01	2.99	0.75	0.00	0.02	0.78	0.00	0.94	0.20	0.84	0.10	2.14	0.04	0.00	0.00	0.00	0.00	0.00	0.05	0.31	0.00	0.04	0.00	0.00	0.22	0.00	0.01	0.00	0.01	0.01	0.01	0.02	0.00	0.00	0.00	0.00
06/03/2013	March	Seston	2.37	0.08	0.59	0.04	5.05	0.92	0.00	0.04	2.09	0.00	1.04	0.15	0.21	0.12	0.21	0.06	0.00	0.00	0.00	0.00	0.00	0.04	0.12	0.00	0.06	0.00	0.00	0.06	0.00	0.04	0.00	0.02	0.04	0.02	0.02	0.00	0.00	0.00	0.00
06/03/2013	March	Seston	1.52	0.03	0.33	0.02	3.79	2.67	0.00	0.02	1.32	0.00	0.69	0.10	0.15	0.07	0.17	0.05	0.00	0.00	0.00	0.00	0.00	0.02	0.09	0.00	0.05	0.00	0.00	0.05	0.01	0.02	0.00	0.01	0.03	0.00	0.03	0.00	0.00	0.00	0.00
02/04/2013	April	Seston	6.21	0.09	1.03	0.05	8.50	0.76	0.00	0.07	1.91	0.00	4.05	0.94	1.31	0.18	1.56	0.14	0.00	0.00	0.00	0.00	0.00	0.09	0.30	0.00	0.07	0.02	0.00	0.25	0.00	0.02	0.00	0.05	0.07	0.09	0.07	0.00	0.00	0.00	0.00
02/04/2013	April	Seston	6.69	0.13	1.82	0.06	10.43	0.84	0.52	0.06	2.39	0.00	3.86	1.25	1.36	0.00	1.49	0.19	0.00	0.00	0.00	0.00	0.00	0.09	0.28	0.02	0.09	0.04	0.00	0.22	0.00	0.04	0.00	0.02	0.06	0.02	0.06	0.00	0.00	0.00	0.00
02/04/2013	April	Seston	1.86	0.04	0.37	0.02	3.77	0.32	0.06	0.03	0.78	0.00	1.31	0.33	0.68	0.00	0.57	0.05	0.00	0.00	0.00	0.00	0.00	0.02	0.12	0.00	0.04	0.01	0.00	0.07	0.00	0.01	0.00	0.01	0.03	0.01	0.02	0.00	0.00	0.00	0.00
02/04/2013	April	Seston	2.32	0.04	0.41	0.02	3.99	0.32	0.00	0.04	0.93	0.00	2.12	0.41	0.71	0.00	0.58	0.05	0.00	0.00	0.00	0.00	0.00	0.03	0.13	0.00	0.04	0.01	0.00	0.07	0.00	0.02	0.00	0.01	0.03	0.04	0.03	0.00	0.00	0.00	0.00
07/05/2013	May	Seston	4.48	0.05	0.31	0.02	4.29	3.31	0.01	0.02	0.89	0.00	2.16	1.63	1.26	0.00	1.84	0.10	0.00	0.00	0.01	0.01	0.01	0.18	0.78	0.00	0.05	0.00	0.00	0.71	0.01	0.02	0.00	0.01	0.02	0.01	0.03	0.00	0.00	0.00	0.00
07/05/2013	May	Seston	5.58	0.09	0.64	0.05	5.89	3.77	0.01	0.03	1.14	0.00	2.92	2.03	1.55	0.13	2.27	0.12	0.00	0.00	0.02	0.01	0.01	0.24	1.00	0.00	0.05	0.00	0.00	0.90	0.01	0.03	0.00	0.02	0.02	0.02	0.04	0.00	0.00	0.00	0.00
07/05/2013	May	Seston	4.04	0.04	0.25	0.01	3.77	0.14	0.01	0.03	0.71	0.00	2.75	1.18	1.46	0.00	2.79	0.10	0.00	0.00	0.01	0.01	0.01	0.09	0.74	0.00	0.05	0.01	0.00	0.94	0.01	0.02	0.01	0.01	0.02	0.01	0.03	0.00	0.00	0.00	0.00
07/05/2013	May	Seston	3.50	0.04	0.24	0.02	3.25	0.12	0.01	0.03	0.55	0.00	2.48	1.02	1.27	0.00	2.39	0.09	0.00	0.02	0.01	0.01	0.01	0.08	0.67	0.00	0.04	0.01	0.00	0.89	0.01	0.02	0.01	0.01	0.02	0.01	0.03	0.00	0.00	0.00	0.00

Figure

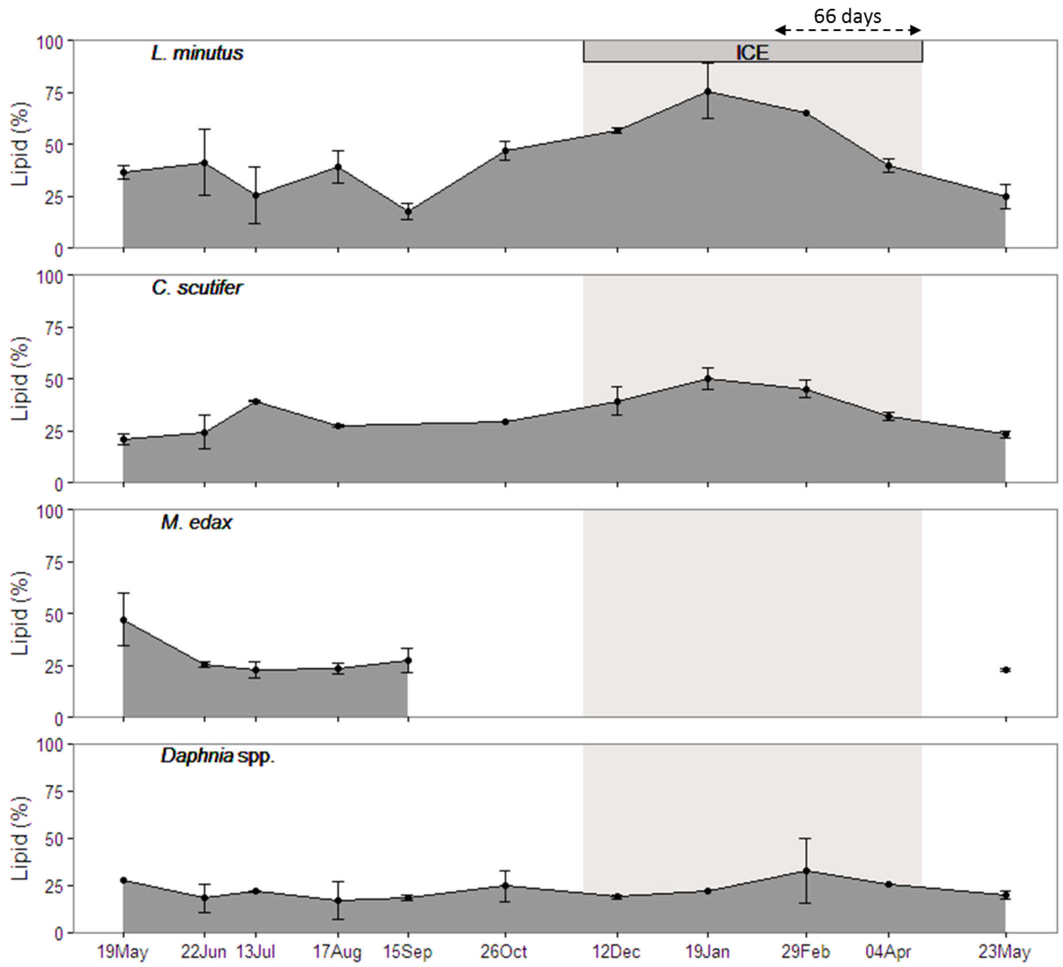

Figure S1
Seasonal pattern of lipid content (%) in 4 different species of zooplankton. Grey shade represents the period when the lake was ice covered. The gap in *M. edax* represents a period of the year when all individuals were absent of the pelagic environment.

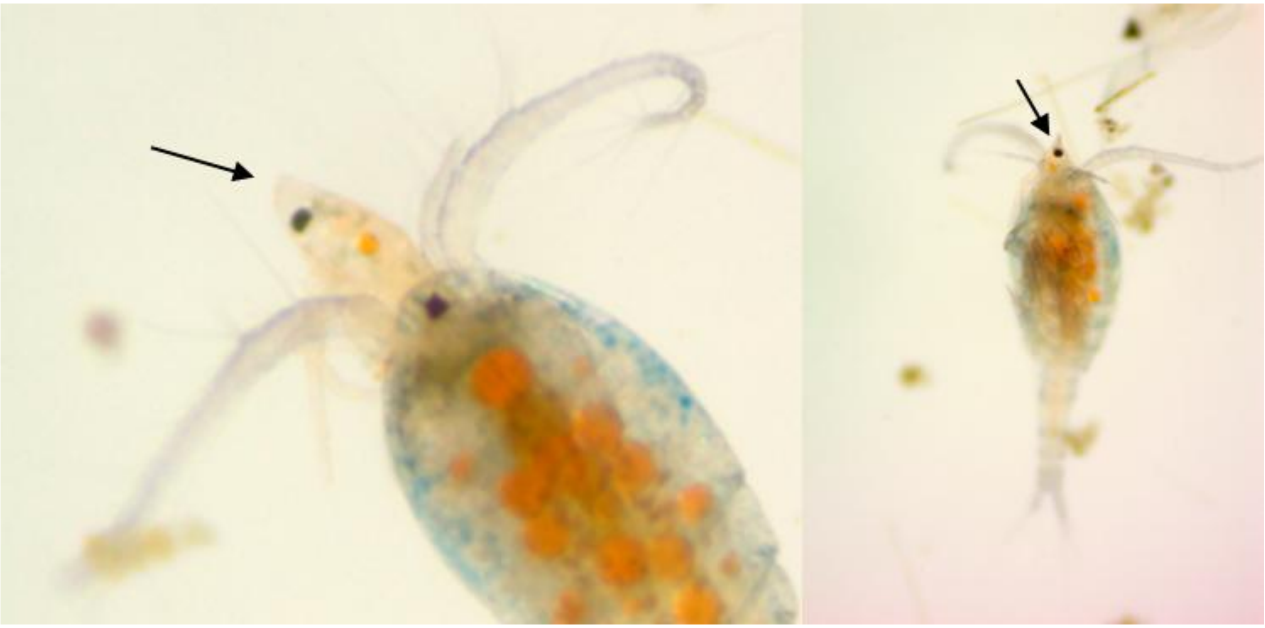

Figure S2
Mesocyclops edax feeding on a *Daphnia* sp. Arrows indicates the prey *Daphnia* sp.

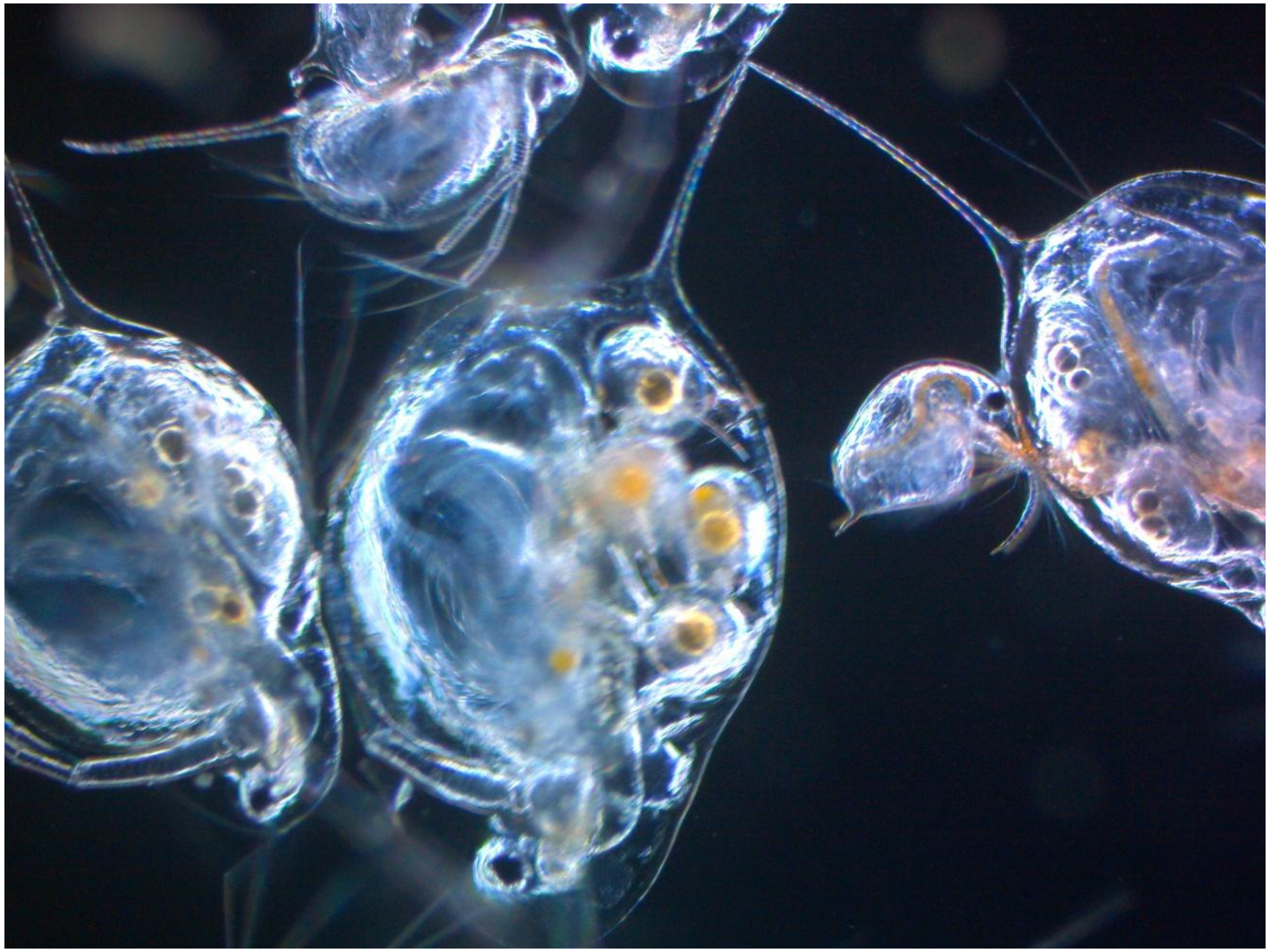

Figure S3

Daphnia spp. with parthenogenetic eggs and young (27 January 2017)
